# Volatile Compounds in Actinomycete Communities: A New Tool for Biosynthetic Gene Cluster Activation, Cooperative Growth Promotion, and Drug Discovery

**DOI:** 10.3390/cells11213510

**Published:** 2022-11-05

**Authors:** Lorena Cuervo, Carmen Méndez, José A. Salas, Carlos Olano, Mónica G. Malmierca

**Affiliations:** 1 Functional Biology Department, University of Oviedo, 33006 Oviedo, Spain; 2 University Institute of Oncology of Asturias (I.U.O.P.A), University of Oviedo, 33006 Oviedo, Spain; 3 Health Research Institute of Asturias (ISPA), 33011 Oviedo, Spain

**Keywords:** *Streptomyces*, volatile compounds, biosynthetic potential, secondary metabolites, rumycins

## Abstract

The increasing appearance of multiresistant pathogens, as well as emerging diseases, has highlighted the need for new strategies to discover natural compounds that can be used as therapeutic alternatives, especially in the genus *Streptomyces*, which is one of the largest producers of bioactive metabolites. In recent years, the study of volatile compounds (VOCs) has raised interest because of the variety of their biological properties in addition to their involvement in cell communication. In this work, we analyze the implications of VOCs as mediating molecules capable of inducing the activation of biosynthetic pathways of bioactive compounds in surrounding Actinomycetes. For this purpose, several strains of *Streptomyces* were co-cultured in chamber devices that allowed VOC exchange while avoiding physical contact. In several of those strains, secondary metabolism was activated by VOCs emitted by companion strains, resulting in increased antibiotic production and synthesis of new VOCs. This study shows a novel strategy to exploit the metabolic potential of Actinomycetes as well as emphasizes the importance of studying the interactions between different microorganisms sharing the same ecological niche.

## 1. Introduction

The pandemic caused by SARS-Cov2 has only highlighted something that has been known for several decades: the lack of new bioactive compounds useful not only to treat diseases caused by emerging pathogens but also to treat those due to pathogens resistant to conventional treatments. The World Health Organization (WHO) declared antimicrobial resistance one of the top 10 global public health threats facing humanity in 2019 and urged the scientific community to focus on new drug research and development [1].

Most anti-infective drugs are of natural origin, mainly produced by bacteria (with special mention to Actinomycetes) and fungi. In natural product research, re-isolation of already known compounds is a major bottleneck, therefore new approaches need to be followed [2,3]. The search for antibiotic producers in underexplored environments or mutualistic relationships with other organisms has demonstrated its usefulness. Marinopyrrole A and abyssomicin C are examples of natural drugs produced by marine bacteria with potent activity against methicillin-resistant *Staphylococcus aureus* (MRSA) [4,5] and polyketides cyphomycin and sipanmycin isolated from the microbiome of leaf-cutter ants of the *Attini* tribe have demonstrated antifungal and antibacterial properties, respectively [6,7].

The development of new genomic techniques has revitalized the field of drug discovery as it has revealed the enormous biosynthetic potential of some microorganisms, such as Actinomycetes. In past years, the sequencing of the genome of a vast amount of Streptomycetes has pointed out the presence of a higher number of biosynthetic gene clusters (BGCs) for the production of secondary metabolites than initially thought. Many of these BGCs are not expressed under laboratory conditions; thus, their products remain unknown. The induction of the expression of these silent BGCs is an attractive research field in drug discovery and efforts have been focused mainly on five approaches: (i) genetic manipulation of global or cluster-specific transcriptional regulators; (ii) expression of natural or synthetic BGCs in native or heterologous hosts; (iii) ribosomal engineering; (iv) OSMAC approaches through the systematic variation of growth parameters; and (v) mimicking the ecological environment of the producer strain via co-culture with other microorganisms or via the use of chemical elicitors (e.g., rare earth elements, dimethyl sulfoxide, ethanol) [8,9,10,11,12,13].

Traditionally, research on natural products was based on water-diffusible compounds, but in recent years, the importance of volatile natural compounds with interesting bioactivities has attracted the attention of the scientific community. Recently, Liu and co-workers applied a machine-learning workflow called MSHub/GNPS [14] to 37 different *Streptomyces* isolates and discovered the production of 581 different volatile compounds, which pointed out the high capacity of this bacterial genus to synthesize this kind of metabolite [15]. Volatile compounds (VOCs) are chemically diverse metabolites with common features: low molecular mass, high vapor pressure, low boiling point, and lipophilic nature [16]. In complex microbial environments, inter- and intra-species relationships are essential to maintain the balance of the community, and secondary metabolites play a key role in this equilibrium, as they participate in signaling, antibiosis, or competition between species [17,18,19,20]. As stated above, bacteria in mutualistic relationships with leaf cutter ants are an important source of new drugs. These ants collect leaves within their nests to cultivate the basidiomycete *Leucoagaricus gongylophorus*, which processes plant material, providing a rich source of nutrients for the ants [21]. This fungal garden is threatened by the pathogenic fungus *Escovopsis weberi,* and to fight against it and protect the stability of the nest, the ants have developed a complex microbiome composed of antimicrobial-producing Actinomycetes (mainly *Pseudonocardia* spp. and *Streptomyces* spp.) [22]. The production of specialized diffusible bioactive metabolites (e.g., candicidin D, antimycins, selvamycin) by these mutualistic bacteria has been reported [23]. In a confined space with many chambers filled with air (as is the case of those nests), it is logical to think that volatile compounds would play a significant role in this war. In 2021, Dhodary and Spiteller described the antifungal properties of inorganic ammonia produced by *Streptomyces* symbionts on *Escovopsis* sp. through the alkalization of the medium [24]. However, what about VOCs? Could they exert direct antibiotic activity or stimulate other microorganisms to produce antibiotics? 

In this work, we tested the hypothesis that VOCs emitted by Actinomycetes isolated from leaf-cutter ants could serve as communication signals capable of activating the production of bioactive compounds by other Actinomycetes within the community. Accordingly, we confronted thirteen different Actinomycete strains isolated from the surface of the *Attini* ants (CS strains [7]) in solid culture using a specialized device designed to study the effect of the VOCs produced in the system (VOC chamber [25]). Using a bioassay-guided screening approach, we detected the activation or overproduction of chemically diverse bioactive natural compounds by Actinomycetes (e.g., cycloheximide, collismycin, cosmomycins, rumycins) when they were exposed to VOCs from other related species. Thus, the important role of volatiles as signaling agents and their usefulness for drug discovery studies were addressed.

## 2. Materials and Methods

### 2.1. Strains and Culture Conditions

The Actinomycete strains used in this work belong to an in-lab CS collection isolated from the cuticle of leafcutter ants from the tribe *Attini* [7,26]. Strains were routinely grown on MA plates [27] and incubated at 28 °C for 7 days. For metabolite production, strains were grown on agar plates of R5A [28], soy flour mannitol (SFM; [29]) or YMA (yeast extract 3 g; malt extract 3 g; peptone 5 g and glucose 10 g per liter). 

### 2.2. Dual-Culture Actinomycetes in VOC Chambers

**Figure 1:**
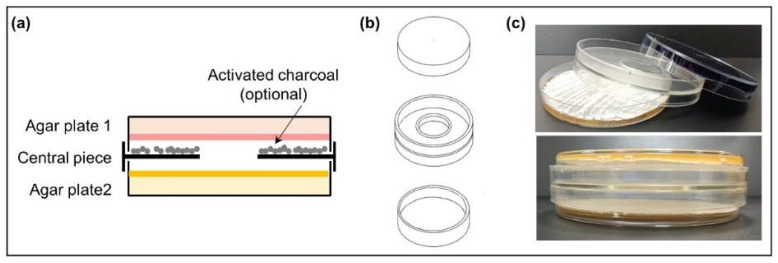
**Volatile compound** (VOC) chamber. (**a**) Schematic side-view of a VOC chamber; (**b**) representation of each part of the VOC chamber device. The hole in the middle allows the exchange of VOCs between cultures (modified from [25]); (**c**) photographs of an assembled VOC chamber (without charcoal).

Actinomycete strains were grown on R5A or SFM plates at 28 °C occupying a surface of 16 cm^2^ in the central part of the plates so that the edges of the colonies could be observed. After 24 h, the VOC chamber (J.D. Catalán S.L., Arganda del Rey, Madrid, Spain) device was mounted as follows: a non-vented central piece was placed on top of the one-day-old growing Actinomycete plate (facing up) and the other plate was placed upside down on top of them (Figure 1). The chamber device has a hole in the central part (without any type of film or filter covering it), allowing the exchange of VOCs between the cultures and avoiding physical contact between the strains or with the compounds that diffuse into the medium. The assembled VOC chamber was sealed with Parafilm^®^ (Bemis, E-Thermo Fisher Scientific, Madrid, Spain). The dual culture was incubated at 28 °C for 5 days. Control VOC chambers were also set with one noninoculated plate and one inoculated plate. Each experiment was made in triplicate.

### 2.3. Multiple Coculture of Actinomycetes

*Streptomyces* sp. CS065a, CS207, CS113, CS149, and CS090a were cultured on YMA or SFM small Petri dishes (diameter of 5 cm) at 28 °C. After 24 h, four opened small plates were placed inside a large Petri dish (diameter of 13,5 cm) and this was sealed with Parafilm^®^ (Bemis, E-Thermo Fisher Scientific, Madrid, Spain). As a control, three noninoculated small plates and the fourth inoculated plate were settled on a large plate. The culture was incubated at 28 °C for 5 days. Each experiment was made in triplicate.

### 2.4. Antibiotic Production in Co-Culture

Agar diffusion bioassays against *Micrococcus luteus* (Gram-positive bacteria), *Escherichia coli* (Gram-negative bacteria), the yeast *Candida albicans*, and the ascomycete *Escovopsis weberi* were performed to test antibiotic production in cocultures. Fresh cultures or fungal spores of each test microorganism were used as seed cultures to inoculate agar plates of TSA (for bacteria), YMA (for yeast), or SFM (for fungus). A 6 mm agar plug from each Actinomycete plate of the cocultures grown for 5 days in the VOC chambers was placed on top of the bioassay plate. The plates were then incubated at 4 °C for one hour to allow metabolites to diffuse into the surrounding medium. Subsequently, the plates were incubated for 16 h at 30 °C (antifungal tests) or 37 °C (antibacterial tests). Agar plugs from control plates grown in single culture were also used. The diameter of the inhibition zones was measured and compared with the control sample. Each test was performed in triplicate.

### 2.5. Extraction of Secondary Metabolites Produced in VOC Chambers, Analysis with UPLC, and Dereplication

We then extracted 2.5 g of the actinomycete agar plates grown in coculture using 3 mL of different organic solvents [ethyl acetate, ethyl acetate containing formic acid (1%) or butanol] and analyzed the extract via reverse phase chromatography in an Acquity UPLC instrument fitted with a BEHC18 column (1.7 μm, 2.1 mm × 100 mm, Waters), with acetonitrile and MQ water + 0.1% trifluoroacetic acid (TFA) as the mobile phase. The PDA detector was set to scan wavelengths between 200 and 600 nm. Samples were eluted with acetonitrile (10%) for 1 min, followed by a linear gradient of acetonitrile (10–100%) for 7 min (flow rate of 0.5 mL/min; column temperature 35 °C). The identity of the metabolites present in these samples was checked via HRMS-based dereplication against MEDINA using an Agilent 1200 Rapid Resolution HPLC coupled with a maXis Bruker qTOF mass spectrometer. The volume injected was 2 µL and a Zorbax SB-C8 column (2.1 × 30 mm, 3.5 µm particle size) was used for separation. The mobile phase consisted of solvent A, 90:10 milliQ water-acetonitrile, and solvent B, milliQ water-acetonitrile, both with 13 mM ammonium formate and 0.01 TFA. Samples were eluted with a 0.3 mL/min flow rate, and the gradient used was 90% to 0% to solvent A/10% to 100% solvent B in 6 min, 0% solvent A/100% solvent B in 2 min, 0% to 90% solvent A/10% to 100% solvent B in 0.1 min, and 90% solvent A/10% solvent B for 1.9 min. The maXis qTOF mass spectrometer was operated in ESI positive mode. Source conditions were 4-kV capillary voltage, end plate offset = 500 V, dry gas (N_2_) flow = 11 L/min; dry temperature = 200 °C, and nebulizer (N_2_) pressure at 2.8 bars. Transfer line conditions were RF 300 Vpp, isCD energy = 0 eV, hexapole = 60 Vpp, quadrupole ion energy = 5 eV, collision cell energy = 10 eV. The mass spectrometer operated with a mass range of m/z 150–2000 and a spectral acquisition rate of 3 Hz. TFA-Na cluster ions were used for mass calibration of the instrument prior to sample injection. Prerun calibration was via infusion with the same TFA-Na calibrant. The retention time, together with the exact mass (and the derived molecular formula), was used as a criterion to search the internal database from Fundación MEDINA [30] and the Dictionary of Natural Products version 26:2 [31] to identify already known compounds.

### 2.6. Purification of Rumycins

Thirty VOC chambers were mounted as described in Section 2.2, placing the strains CS149 and CS131 cultured on SFM and incubated at 28 °C for seven days across from each other. The thirty plates in which the CS149 strain was grown were extracted with 600 mL of butanol and subsequently filtered, concentrated under vacuum and resuspended in MQ water. The sample was fractionated through a 10 g Sep-PaK^®^ Vac 35 cc C18 cartridge (Waters) using as mobile phase solvent methanol: MQ water at 5 mL/min and a gradient of 0% to 100% methanol for 55 min. 

Purification of the desired fractions was carried out via reverse phase chromatography on an Alliance HPLC chromatographic system (Waters 2695 Separation Module) coupled to a Waters 996 Photodiode Array Detector, using a Sunfire C18 column (10 µm, 10 mm × 280 mm, Waters) and an isocratic mixture 55:45 MQ Water: ACN for rumycin 1 and 20:80 for rumycin 2 at a flow rate of 5 mL/min. The compounds were collected and lyophilized.

### 2.7. Biosynthetic Gene Cluster Prediction and Sequence Analysis

Biosynthetic gene cluster prediction for secondary metabolite searches and sequence analysis was carried out with the online bioinformatic tool antiSMASH v6 [32]. Rumycin gene clusters were deposited at the Minimum Information about a Biosynthetic Gene Cluster (MIBiG) repository [33] under the accession number BGC0002753.

## 3. Results and Discussion

### 3.1. Morphological and Developmental State of Actinomycete strains in VOC Chambers

The morphology of strains growing on R5A, SFM, and YMA agar plates was compared in monoculture versus coculture in VOC chambers. No changes in growth rate were detected and only a slight difference was observed in the timing of the sporulation stage depending on the particular strain and the strain with which it was paired. In addition, exploratory behavior at the edge of the colonies was evaluated as a previous study had revealed the importance of VOCs in this kind of development (Figure S1, Supplementary data). Fungal VOCs triggered exploratory growth in *Streptomyces venezuelae* colonies, and these “activated” cells could induce this developmental state in other physically separated *Streptomyces* colonies by producing the airborne compound trimethylamine (TMA) [34]. In the present study, exploratory growth was not observed in any of the tested strains. This result could be due to the lack of TMA production by the CS strains or by a different response to this VOC by the Actinomycete strains employed in the present assay. Further work is ongoing trying to clarify this point.

**Figure 2:**
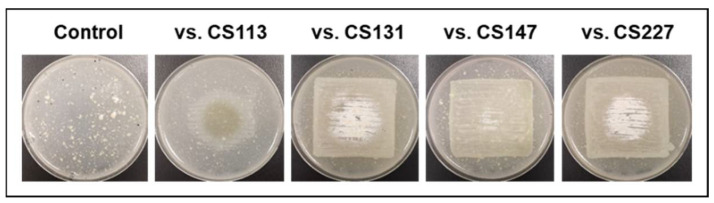
Strain CS194 grown in SFM medium paired with different CS strains in VOC chambers.

The only exception to the observations was *Streptomyces* sp. CS194. When cultured on SFM medium, this strain was unable to grow in monoculture (control). Only when exposed to VOCs from other CS strains did CS194 reach different levels of development (Figure 2). This fact highlights the importance of VOCs in the communication between strains within a complex community and demonstrates their role as growth-promoting agents. Traditionally, microbial growth and development studies have been mainly focused on pure-culture systems, but now there is increasing evidence that cannot be avoided: microbes live in changing systems of multiple species, and for that reason, the interaction between them should be taken into account to gain a deeper understanding of microbial physiology [35,36]. Several studies have referred to the developmental changes induced by water-diffusible compounds. The siderophore desferrioxamine E enhanced growth and antibiotic activity in several *Streptomyces* species as well as the production of goadsporin, a microcin-like peptide [37,38]. In addition, volatile γ-butyrolactones have been described as quorum sensing molecules that stimulate aerial growth and metabolite production in *Streptomyces* [39,40]. Most of the strains that promoted the growth and development of strain CS194 presented in their genomes BGCs responsible for the synthesis of different types of butyrolactones [41], thus the growth-promoting effect observed in this work could be due to this family of compounds. More experiments need to be performed to elucidate this point.

### 3.2. New or Incremental Increases in Antibiotic Production in VOC Chambers

**Figure 3:**
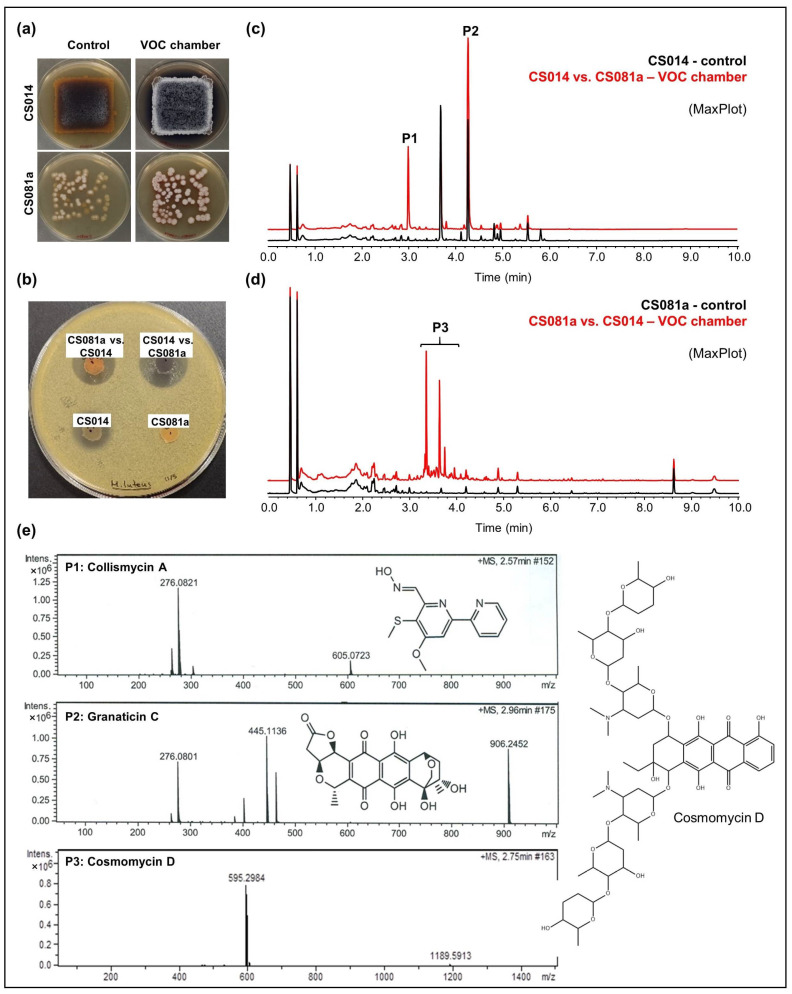
Overview of the results obtained for the strains CS014 and CS081a grown on YMA medium. (**a**) Upper-view photographs of YMA plates where the strains were grown alone (control plates) or co-cultured in a VOC chamber; (**b**) bioassay plate against *Micrococcus luteus*; (**c**,**d**) UPLC chromatograms (MaxPlot) of samples extracted with ethyl acetate. Peaks observed only (or in a higher amount) in co-culture were highlighted; (**e**) HRMS spectra and chemical structures of the related compounds.

**Figure 4:**
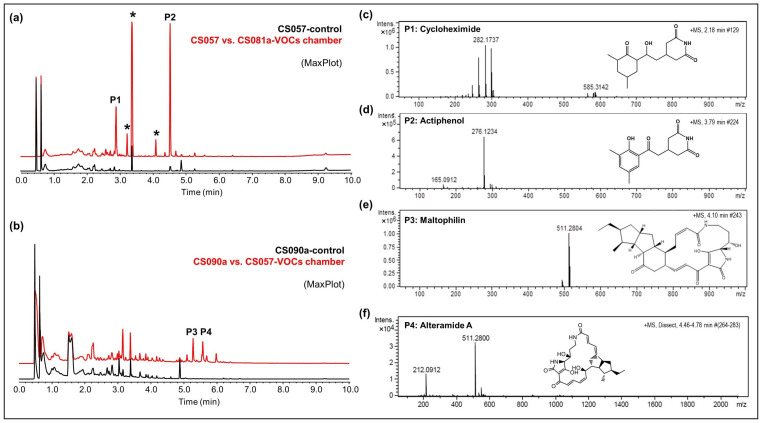
Activation of secondary metabolism in CS057 and CS090a strains. (**a**) Comparative UPLC analysis of samples of CS057 (control plate, black) and CS057 exposed to VOCs produced by CS081a (red). Asterisks indicate intermediates of actiphenol biosynthesis; cycloheximide (P1); actiphenol (P2). (**b**) Comparative UPLC analysis of samples of CS090a (control plate, black) and CS090a exposed to VOCs produced by CS057 (red). Maltophilin (P3) and alteramide A (P4); (**c**) HRMS spectrum of cycloheximide, (**d**) HRMS spectrum of actiphenol, (**e**) HRMS spectrum of maltophilin; (**f**) HRMS spectrum of alteramide A.

**Figure 5:**
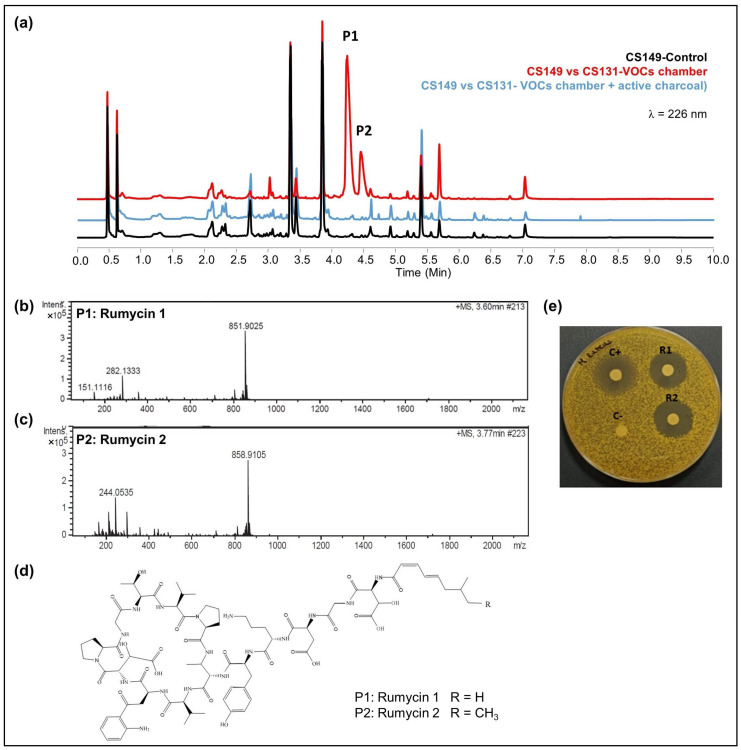
Activation of the synthesis of rumycins by the effects of VOCs from *Streptomyces* sp. CS131 on CS149. (**a**) Comparative UPLC profile where the peaks containing rumycin 1 and rumycin 2 have been numbered as P1 and P2, respectively; (**b**) HRMS spectrum of rumycin 1; (**c**) HRMS spectrum of rumycin 2. (**d**) Chemical structure of rumycins. (**e**) Bioassay of purified rumycins against *M. luteus*. (C^+^): apramycin (200 mg/mL), (C^−^): methanol, (R1): rumycin 1 (10 mg/mL), (R2): rumycin 2 (10 mg/mL).

Agar plate bioassays were carried out using samples extracted from the CS strains grown in VOC chambers and inhibition areas were compared to controls (strains grown in monoculture). No positive results were obtained when strains were cultivated on R5A and only strains CS149 and CS194 showed increased antibiotic activity in coculture when grown on SFM medium. On the contrary, the secondary metabolism of many more strains was activated by VOCs emitted by other colonies on YMA. The results obtained could be summarized as follows (complete results are shown in Supplementary Materials Tables S1–S13; Data concerning the characterization of compounds by LC-MS are available in Supplementary Material file: LC-MS dereplication):

- CS014: An increased antibiotic activity against *M. luteus* was detected in coculture with CS057, CS081a, CS090a, CS131, and CS149 strains grown on YMA plates (Figure 3a,b). Comparative UPLC analysis revealed the activation of collismycin production in the presence of VOCs from the strains mentioned above. Furthermore, increased production of granaticin C was observed (Figure 3c).
- CS081a: This strain only demonstrated anti-*M. luteus* activity when grown on YMA medium in VOC chambers cultured against CS014 strain (Figure 3b). The chromatographic analysis of samples extracted with ethyl acetate exposed the biosynthetic induction of the cosmomycin anthracycline antibiotic family by CS081a under the effect of CS014 VOCs (Figure 3d). In this case, the signaling caused by volatiles emitted by the strains inside the chamber was bidirectional as VOCs from CS014 impacted the secondary metabolism of the CS081a strain and vice versa (see the previous paragraph).

- CS057: The strain growing on YMA agar plates in a VOC chamber together with CS081a demonstrated stronger bioactivity against *M. luteus* and *E. weberi*. UPLC analysis revealed an increased production of the related compounds cycloheximide and actiphenol by CS057 exposed to VOCs from CS081a (Figure 4a,c,d).
- CS090a: Antifungal compounds active against *E. weberi* were only produced by the CS090a strain when it was grown on YMA under the effect of VOCs originating from CS057 and CS081a. Chromatographic analysis found maltophilin and alteramide activation of the production (Figure 4b,e,f).

- CS149: No antibiotic activity against the tested microorganisms was detected when CS149 was grown in monoculture. However, when paired with CS081a or CS131 in VOC chambers, potent bioactivity against *M. luteus* was observed. Chromatographic analysis highlighted the activation of the production of two different compounds that were not present in samples extracted from monocultures of CS149 (Figure 5a). These compounds were identified by dereplication as the anti-MRSA cyclic lipopeptides rumycin 1 and rumycin 2 (Figure 5b–d). In a step-forward study to confirm that the observed alteration of the CS149 secondary metabolism was due to the VOCs emitted by CS131, a VOC chamber with 2 g of activated charcoal was assembled and then the samples extracted with ethyl acetate from SFM CS149-CS131 dual cultures were analyzed by UPLC. No rumycins could be detected (Figure 5a) indicating that VOCs from CS131 were adsorbed by the activated charcoal, and thus could not exert their inductive effect on CS149 biosynthetic machinery. On the other hand, biosynthesis of rumycins by *Streptomyces* sp. CS149 does not depend strictly on the growth medium of the strain; this has been verified in both SFM and R5A. Otherwise, the VOCs that induced the activation of the secondary metabolism of *Streptomyces* sp. CS149 were only produced by CS081a or CS131 if grown on SFM since rumycins could not be detected when the VOC-emitting strain was cultured on R5A or YMA. We purified 3.6 mg of rumycin 1 and 2.8 mg of rumycin 2 from thirty plates (total volume of 600 mL) of SFM (dual cultures between CS149 and CS131 strains in VOC chambers). Pure compounds were used for the testing of bioactivity via agar diffusion bioassay. They demonstrated potent antibacterial activity against *M. luteus*, even stronger than the commonly used antibiotic apramycin, but no activity against *E. coli*, *C. albicans*, and *E. weberi* (Figure 5e).

- CS194: When cultivated on SFM, the strain CS194 exposed to VOCs emitted by strains CS014, CS057, CS081a, or CS227, presented antibiotic activity versus *M. luteus*. As mentioned previously, this strain was not able to grow on SFM in monoculture, thus we could not state if the production of the antibiotic/s was due to a signaling effect of VOCs on growth promotion or by the induction of the CS194 secondary metabolism (or both). In any case, the antibacterial activity of CS194 could be explained by the production of bioactive piperazinediones, detected by dereplication in samples of CS194 (Figure S2, Supplementary data).
- CS207: The antibacterial (against *M. luteus* and *E. coli*) and antifungal activities of the strain CS207 were higher when cultivated on YMA in VOC chambers together with strains CS014, CS057, CS081a, or CS227. Unfortunately, the comparative UPLC analysis did not retrieve any differential peak that could explain the observed antibiotic activity, probably due to the limitation of the analytics based on UV absorbance measurements.

The close relationship between leaf cutter ants and Actinomycetes has been extensively studied for decades [22,23,42]. Although the true symbiont of the *Attini* ants has been identified as *Pseudonocardia* sp. [43], the role of the Streptomycete population isolated from the surface of those insects has not been fully elucidated. Batey and colleagues reviewed the involvement of these Streptomycetes in protecting the nests from pests using the production of specialized antimicrobial compounds [23]. Thus, symbiotic, mutualistic and antagonistic behaviors take place in the confined spaces delimited by the subterranean nest chambers, in such a way that a controlled network of interspecific communication signals plays a key role in maintaining the stability of the community.

In this work, we have demonstrated the potential of the CS strains as antibiotic producers but, more importantly, their ability to modulate the biosynthetic machinery of other related species. VOCs emitted by these strains provoke the overproduction of compounds with diverse chemical structures known for their remarkable antibiotic activity, as is the case of the benzoisochromanequinone polyketide granaticins [44], the polycyclic tetramate macrolactam alteramides [45], and the glutarimide-containing polyketide family of cycloheximide and actiphenol [46,47], synthesized by *Streptomyces* sp. CS014, CS090a, and CS057, respectively. 

Furthermore, the application of these VOCs in activating silent biosynthetic gene clusters that could lead to the discovery of new compounds with potential biomedical uses is very promising and could be implemented as a routine technique to carry out during drug screening research programs. Interspecific communication between different Actinomycetes mediated by VOCs induced the production of several bioactive compounds that were not biosynthesized when the strains were grown in monoculture. Within the VOC chambers, we observed the induction of the biosynthesis of collismycins (cytotoxic and antibiotic 2,2′-bipyridyl class of compounds [48]), cosmomycins (glycosylated anthracyclines with antibiotic properties [49]), maltophilins (macrolactams with antifungal activity [50]), alteramides, and rumycins (cyclic lipopeptides with strong antibacterial properties, proposed as a treatment against methicillin-resistant *Staphylococcus aureus* infections [51]). The potent bioactivity of rumycins against *M. luteus* observed during this work makes them good candidates for further research on their biosynthesis. 

Notably, the same biosynthetic machinery was activated by VOCs from different strains (e.g., collismycins and granaticins were produced by *Streptomyces* sp. CS014 when exposed to VOCs from five different strains, CS057, CS081a, CS090a, CS131, and CS149). This fact could indicate a common mechanism among Actinomycetes to modulate the secondary metabolism of other related bacteria. One possible explanation could be the production of the same VOC by different strains that triggered the expression of one specific BGC. On the contrary, another plausible mechanism could be the manifestation of the same response of the induced metabolic machinery to different VOCs.

### 3.3. Effect of VOCs in Multiple Co-Culture

**Figure 6:**
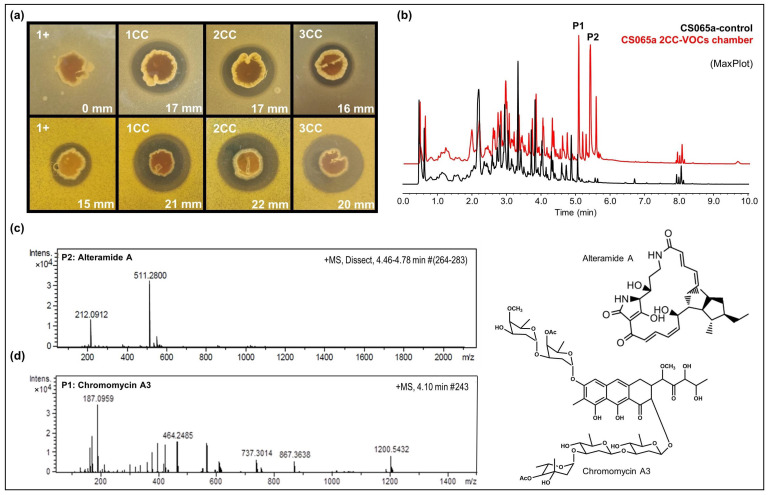
Results of multiple co-culture of CS065a on SFM. (**a**) Bioassay against *E. coli* (first row) and *M. luteus* (second row). 1^+^: *Streptomyces* CS065; 1CC: co-culture CS065a, CS113, CS147, and CS207; 2CC: co-culture CS065a, CS090a, CS147 and CS207; 3CC: coculture CS065a, CS090a, CS113 and CS207. (**b**) Comparative UPLC analysis (MaxPlot) of samples of CS065a in monoculture (black) versus in co-culture with CS090a, CS147, and 207 (red). Differential peaks are marked with an asterisk. (**c**) HRMS spectrum of alteramide A. (**d**) HRMS spectrum of chromomycin A3.

There were some strains in which we could not detect any modification in their capacity to produce antibiotic compounds after being exposed to VOCs from other Actinomycetes in VOC chambers. With those strains, we performed a multiple co-culture approach where the strains were under the effect of VOCs from other three different strains to mimic the complex environment found inside the nests inhabited by the leaf-cutter ants (complete results were shown in Supplementary Materials Tables S14–S18). Applying this approach, we were able to detect higher antibacterial activity against *M. luteus* and *E. coli* in *Streptomyces* sp. CS065a when exposed to VOCs from different combinations of CS strains (Figure 6a). The comparative UPLC profile between samples of *Streptomyces* sp. CS065a grown in monoculture or in multiple cocultures with CS113, CS147, and CS207 (co-culture 1; 1CC); CS090a, CS147, and CS207 (co-culture 2; 2CC; Figure 6b); or CS090a, CS113 and CS207 (co-culture 3; 3CC) revealed activation of the production of several compounds of the alteramide and chromomycin families (Figure 6c,d). The antimicrobial activity against Gram-positive bacteria could be explained by the action of these two groups of compounds [45,52]. Therefore, the observed bioactivity against *E. coli* might in addition be due to the production of one or more compounds that could not be detected under our experimental techniques, since alteramides and chromomycins have not been described as anti-Gram-negative agents individually.

Coculture has been successfully applied in new drug screening programs because it is capable of mimicking interaction between naturally occurring microbial communities. By co-culturing different bacterial and fungal species, the induction of many compounds (e.g., aminoglycosides, terpenes, polyketides, or alkaloids) has been reported [53]. Most of these studies were based on mixed fermentation or solid medium co-cultures, so it is impossible to determine whether the observed metabolic changes are due to water-diffusible or volatile compounds. We show the importance of VOCs in the metabolic modulation of related species and point out the idea that more than one VOC producer may be needed to obtain the desired results. The biosynthesis of alteramides and chromomycins by *Streptomyces* sp. CS065a only occurred when the strain was co-cultured with three other strains, as we were unable to detect those compounds in samples from monoculture or dual-culture using VOC chambers pairing the same strains.

### 3.4. Identification of the Rumycin Biosynthetic Gene Cluster

**Figure 7:**
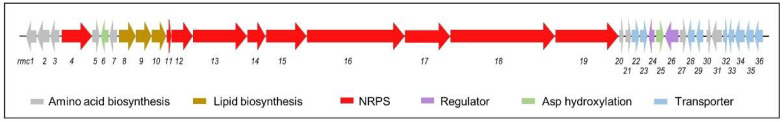
Rumycin (*rmc*) biosynthetic gene cluster.

Among the metabolites whose biosynthetic pathways were activated during this work, only rumycins were not linked to a previously described BGC. Therefore, bioinformatic analysis was carried out to identify the rumycin (*rmc*) gene cluster within the genomic sequence of *Streptomyces* sp. CS149. AntiSMASH v6 predicted the presence of 31 BGCs in the genomic DNA of *Streptomyces* sp. CS149 [41]. Based on the chemical structure of rumycins (cyclic lipopeptides made up of 14 amino acid residues), cluster 17 was identified as the unique candidate for the BGC responsible for the synthesis of rumycins. The *rmc* BGC was classified as an 84 Kb non-ribosomal peptide (NRP) cluster type with a 66% similarity to the cadaside BGC (Figure 7). BLASTp analysis of each *rmc* gene product revealed the presence of genes involved in the synthesis of the NRP chain, transport, regulation, and synthesis of nonproteinogenic amino acids and the acyl chain. A detailed description of the predicted functions of the *rmc* genes is summarized in Table S2 (Supplementary Materials Table S19). 

## 4. Conclusions

In complex microbial ecosystems, communication between individuals is a crucial factor for the survival and health of the community. Inter- and intra-kingdom signaling plays a key role in the spatial and temporal coordination of cellular developmental processes, contributing to the detection of nutritional stress or activating competitive behaviors through antibiosis [54]. Among the wide array of metabolites produced by microorganisms, the chemical properties of VOCs make them the perfect form of communication in an environment full of air gaps such as soil [55]. In this work, VOC chambers have been successfully applied to study the effect of volatiles (separately from water-diffusible compounds) on the secondary metabolism of related species. The production of bioactive compounds with different chemical natures and target microorganisms has been improved by the signaling effect of VOCs emitted by nearby bacterial strains. In addition to its role in antibiosis, a role in modulating the behavior of the bacterial community to better combat pests could be attributed to the actinomycete microbiome of the leaf-cutting ants. As far as we are concerned, this is the first time the VOC-induced production of bioactive compounds by Actinomycetes has been described, pointing out the potential of volatile compounds as a useful tool for drug discovery.

## Data Availability

The data presented in this study are available on request from the corresponding author.

## Supplementary Materials

The following supporting information can be downloaded at: https://www.mdpi.com/article/10.3390/cells11213510/s1, LC-MS dereplication: Data corresponding to the identification of compounds whose production was activated in this work. Figure S1: Complete panel of dual cultures installed in VOC chambers pairing *Streptomyces* sp. CS057 with the rest of the CS strains. Top and bottom views of the YMA agar plates on day 5 of the co-culture. Figure S2: Dereplication analysis of a sample of the CS194 strain grown in SFM medium under the effect of VOCs emitted by CS227. Tables S1–S13: Overview of the complete results obtained in dual co-cultures using VOC chambers. Tables S14–S18: Overview of the complete results obtained in multiple cocultures using VOC chambers. Table S19. Predicted function of *rmc* genes.
