# Supplementary material for: Volatile Compounds in Actinomycete Communities: A New Tool for Biosynthetic Gene Cluster Activation, Cooperative Growth Promotion, and Drug Discovery
Source: Cells. 2022 Nov 5;11(21):3510. doi: 10.3390/cells11213510 (PMC9655753; doi:10.3390/cells11213510)
Supplement: Supplementary file 1 [file cells-11-03510-s001.zip › LC-MS dereplication.pdf]

LC-MS dereplication.

Section 3.2. New or incremented antibiotic production in VOC chambers

CS014:

Collismycin A-B, $C_{13}H_{13}N_{3}O_{2}S$:

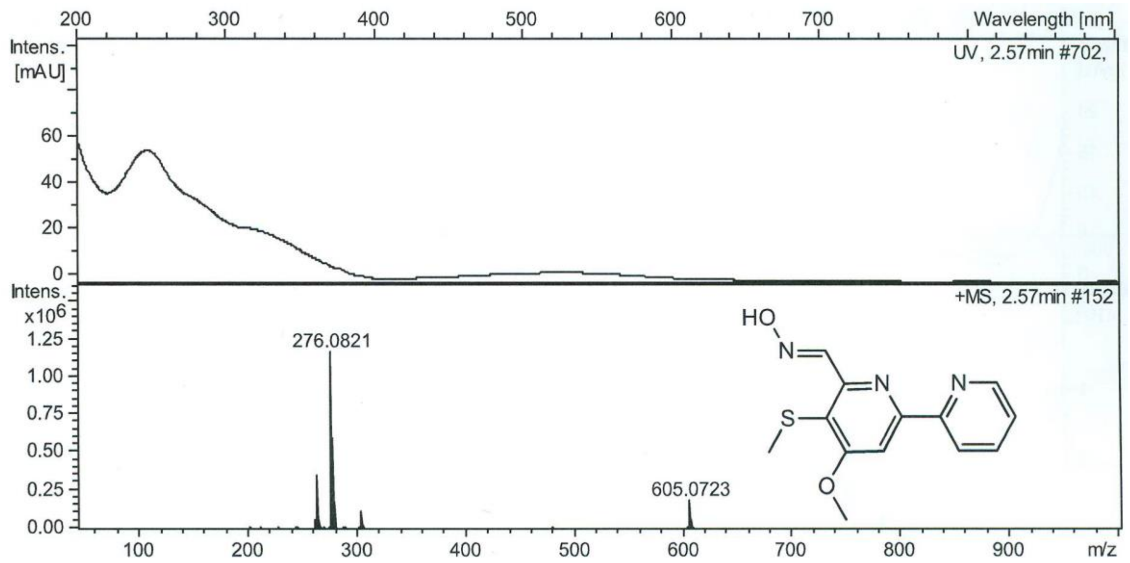

Collismycin C, $C_{13}H_{14}N_{2}O_{2}S$:

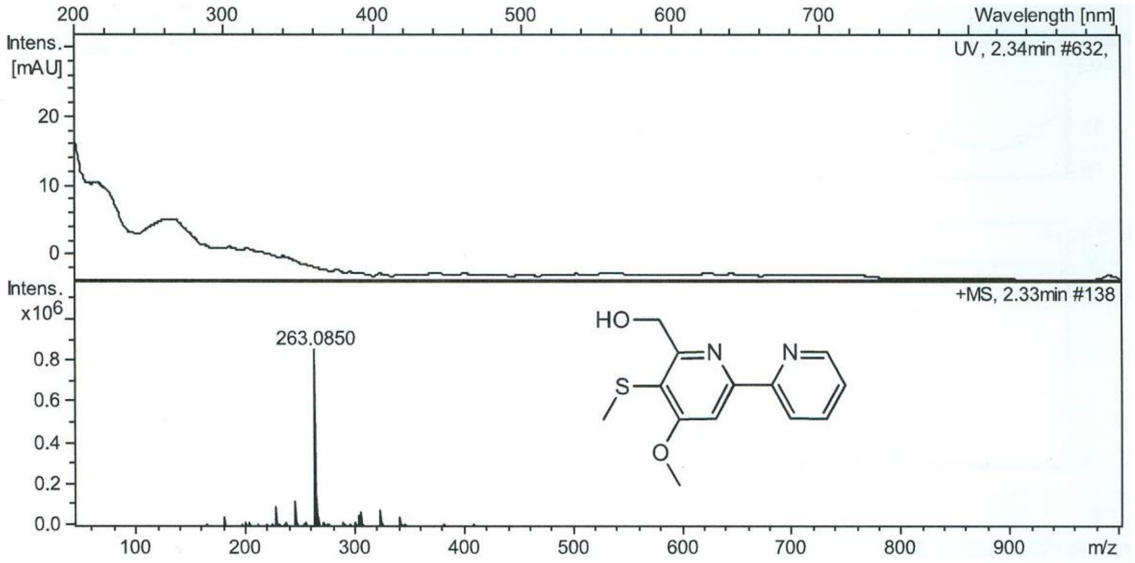

Collismycin D, $C_{13}H_{11}N_3OS$:

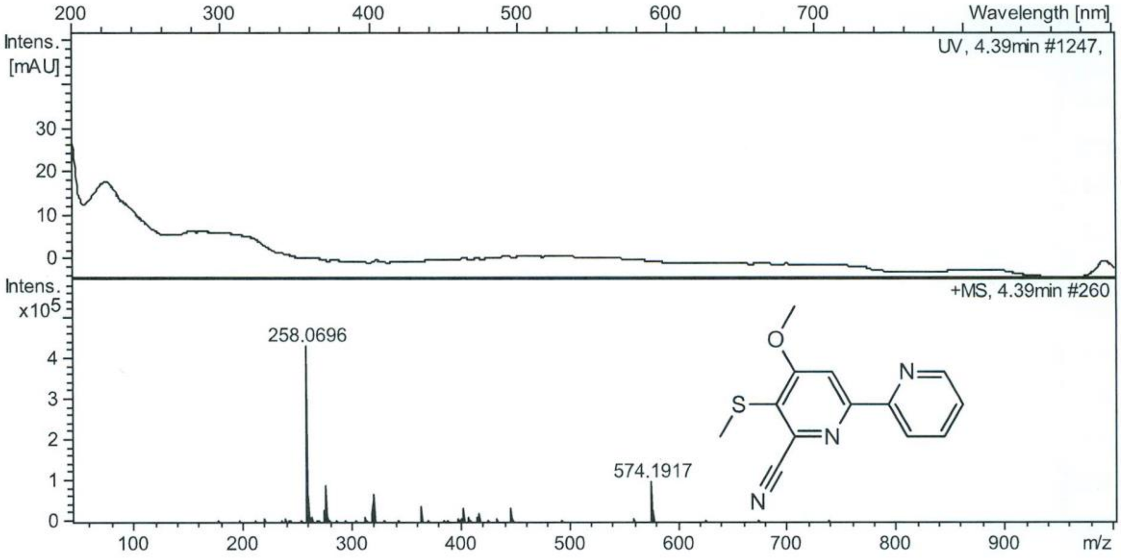

Granaticin A, $C_{22}H_{20}O_{10}$:

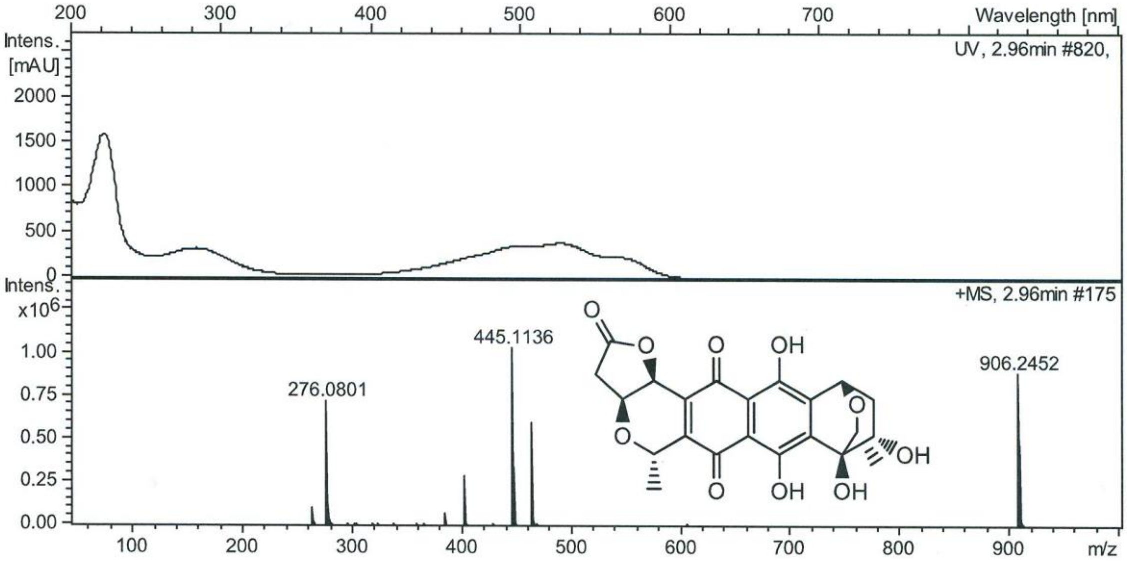

Granaticin C, $C_{28}H_{30}O_{12}$:

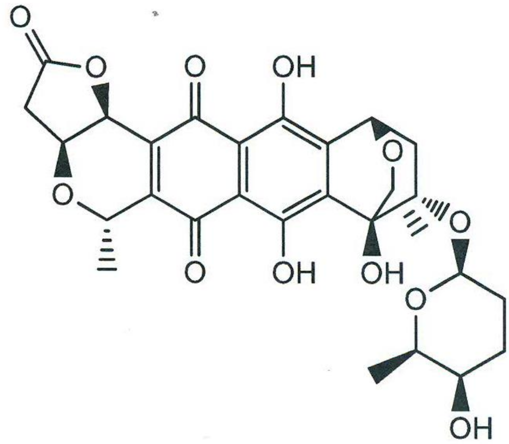

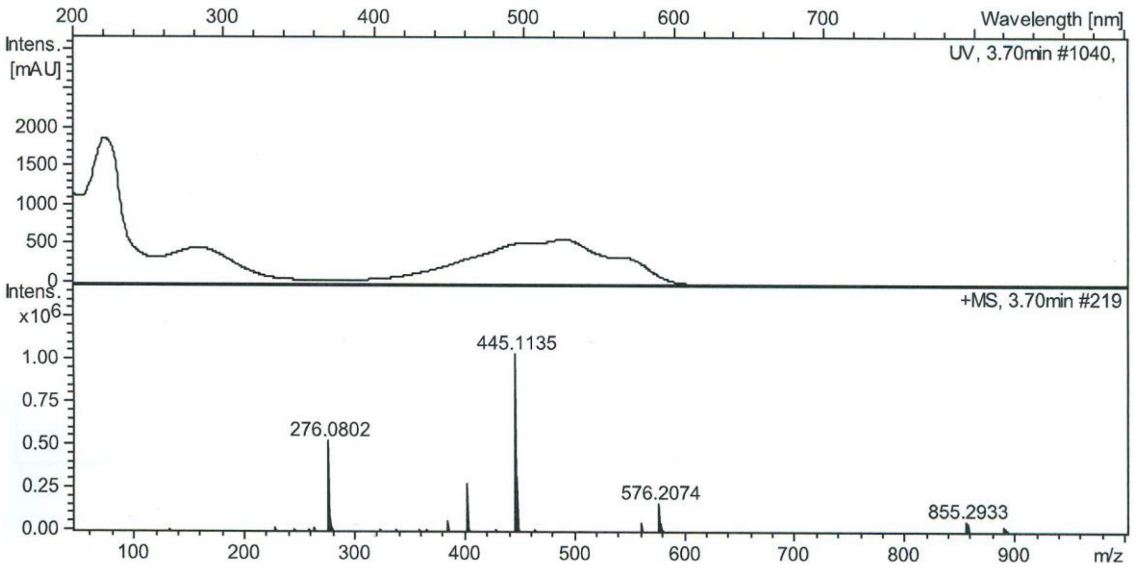

CS081a:

Cosmomycin D, $C_{60}H_{88}N_2O_{22}$:

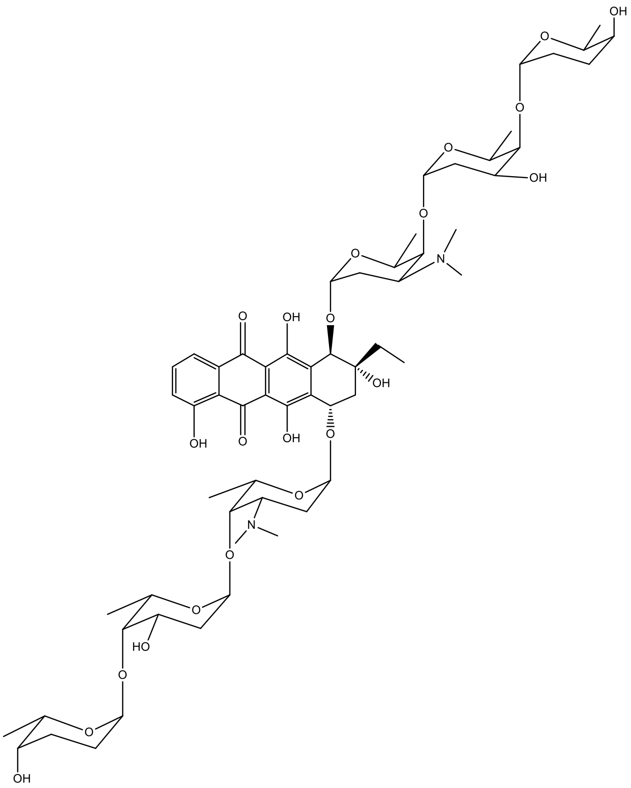

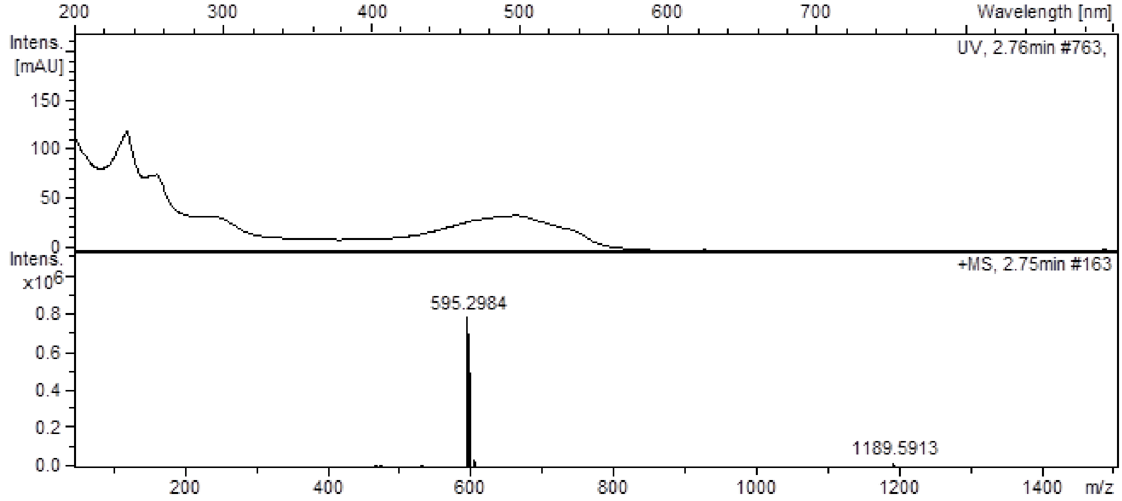

CS057:

Cycloheximide, $C_{15}H_{23}NO_4$:

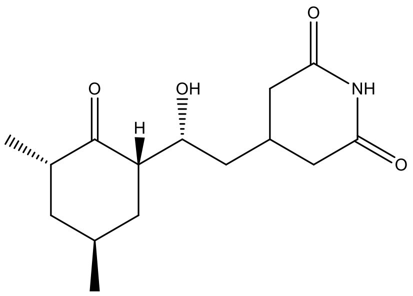

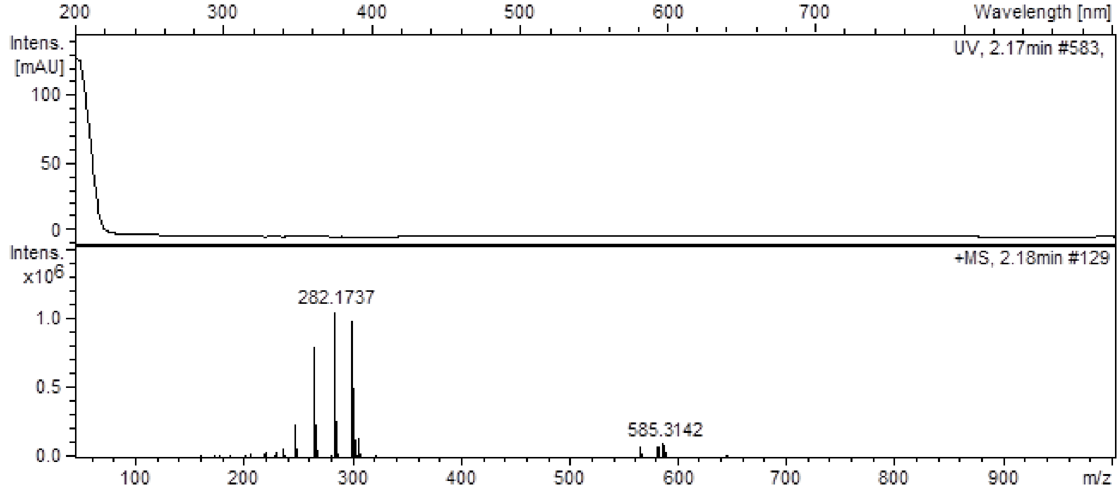

Actiphenol, $C_{15}H_{17}NO_4$:

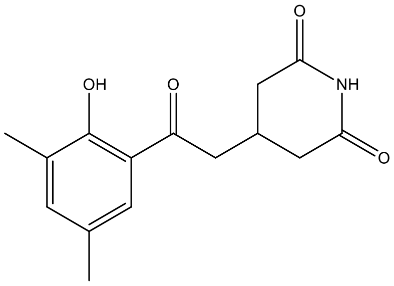

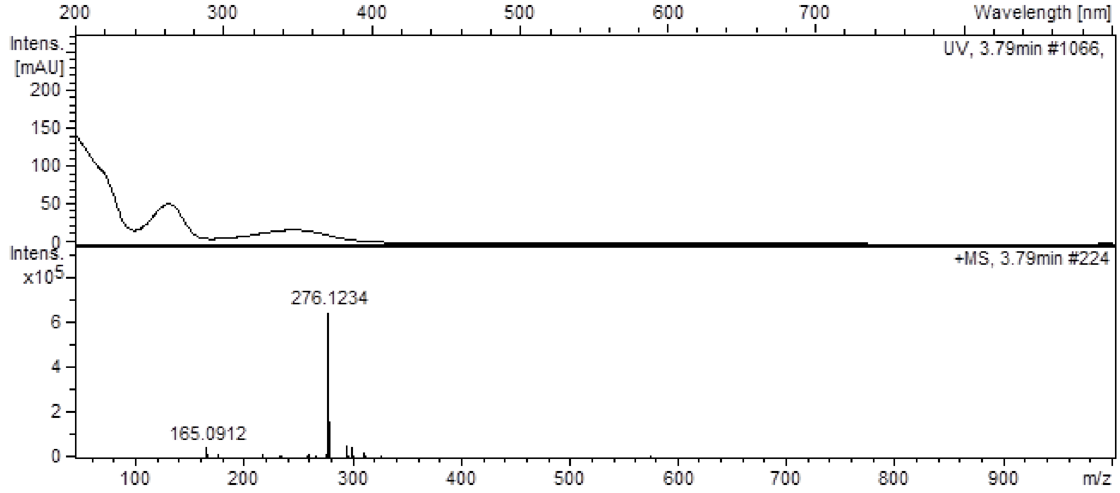

CS090a:

Maltophilin, $C_{29}H_{38}N_2O_6$:

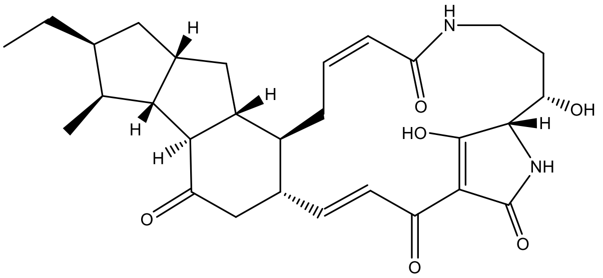

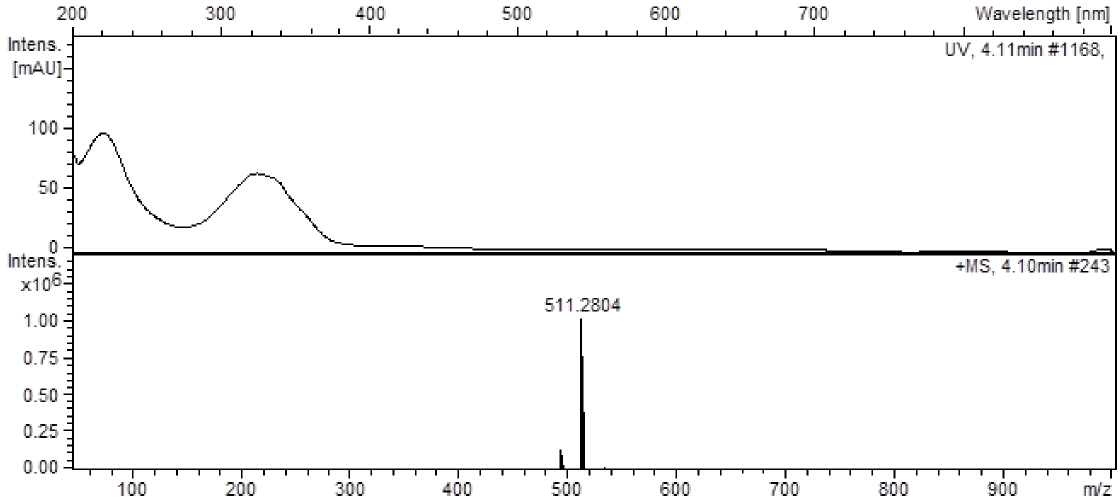

Alteramide A, $C_{29}H_{38}N_2O_6$:

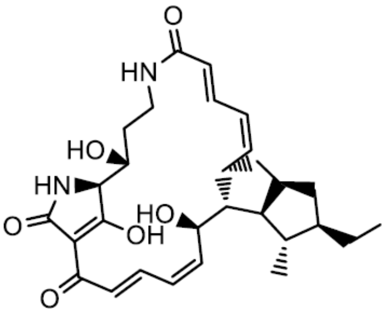

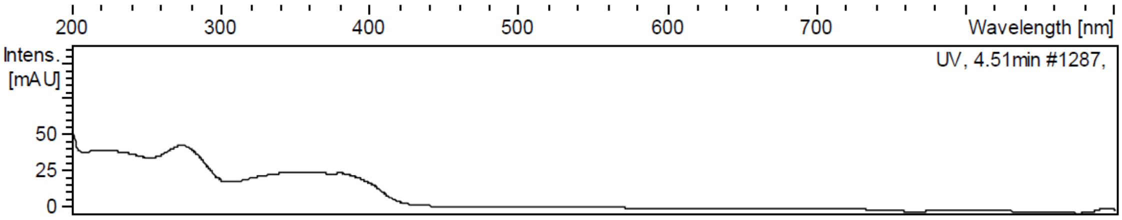

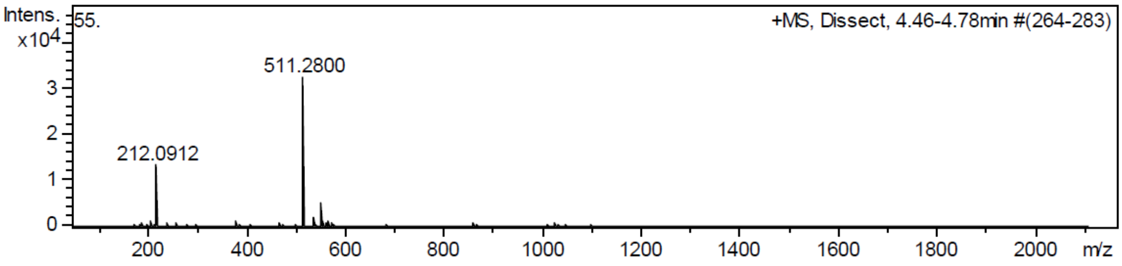

CS194:

Piperazinedione XR 334, $C_{19}H_{16}N_2O_3$:

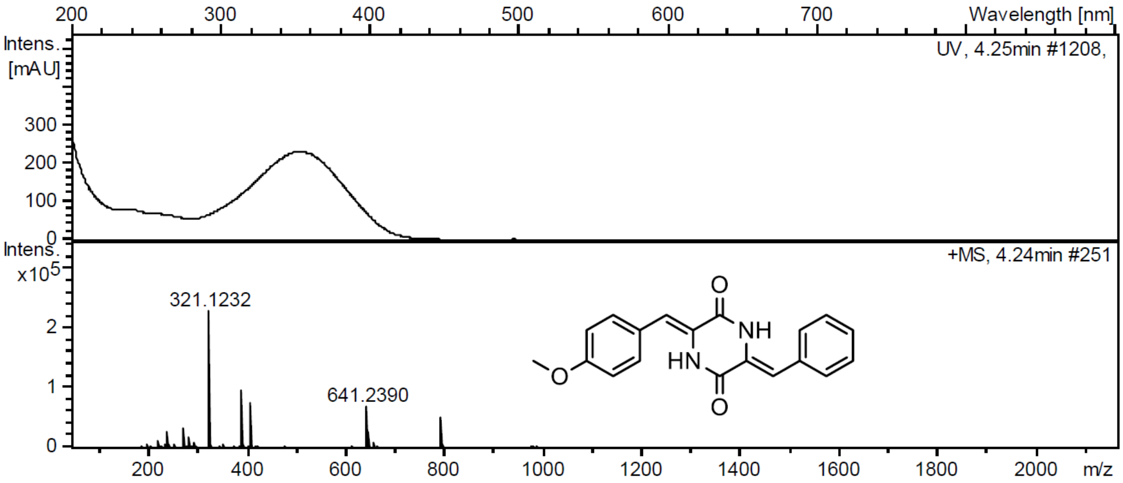

Piperazinedione XR 330, $C_{20}H_{18}N_2O_3$:

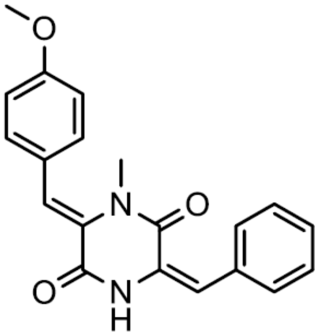

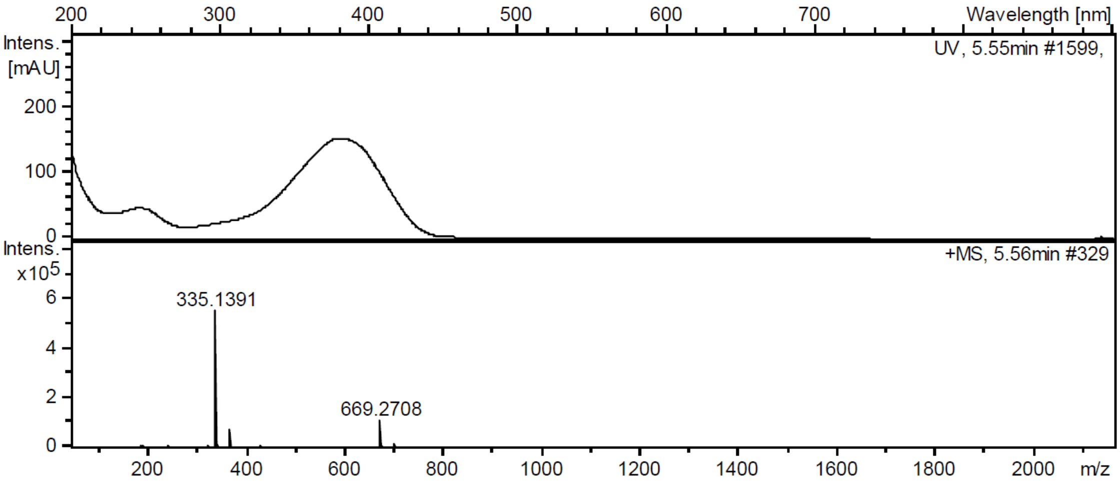

CS149:

Rumycin 1, $C_{78}H_{111}N_{17}O_{26}$:

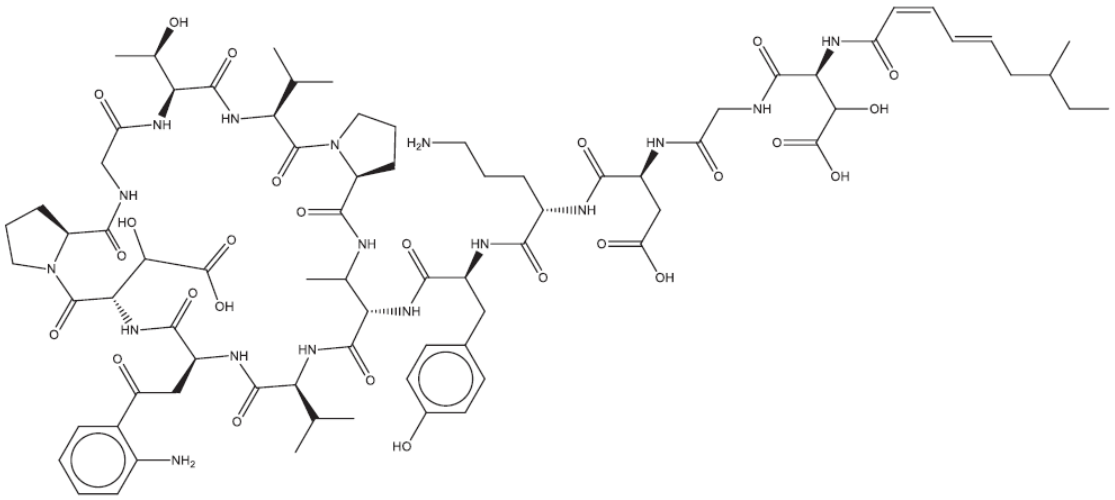

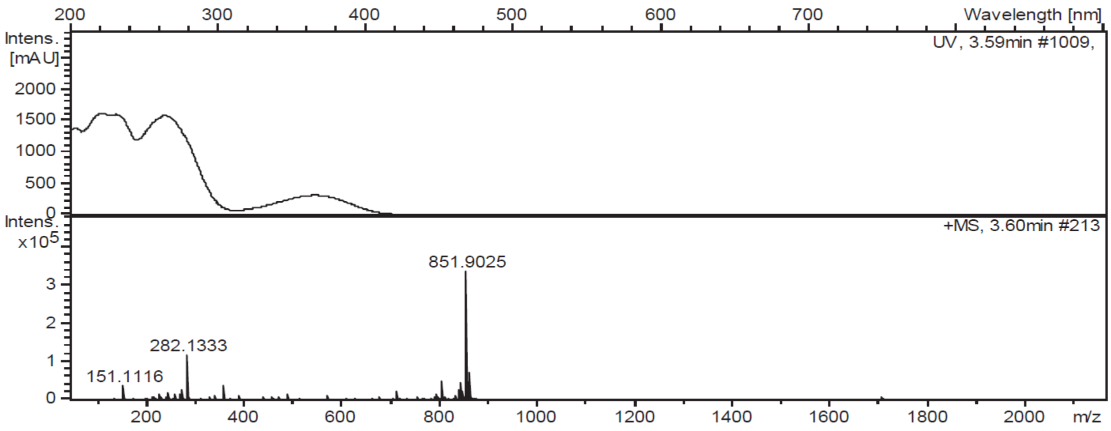

Rumycin 2, $C_{79}H_{113}N_{17}O_{26}$:

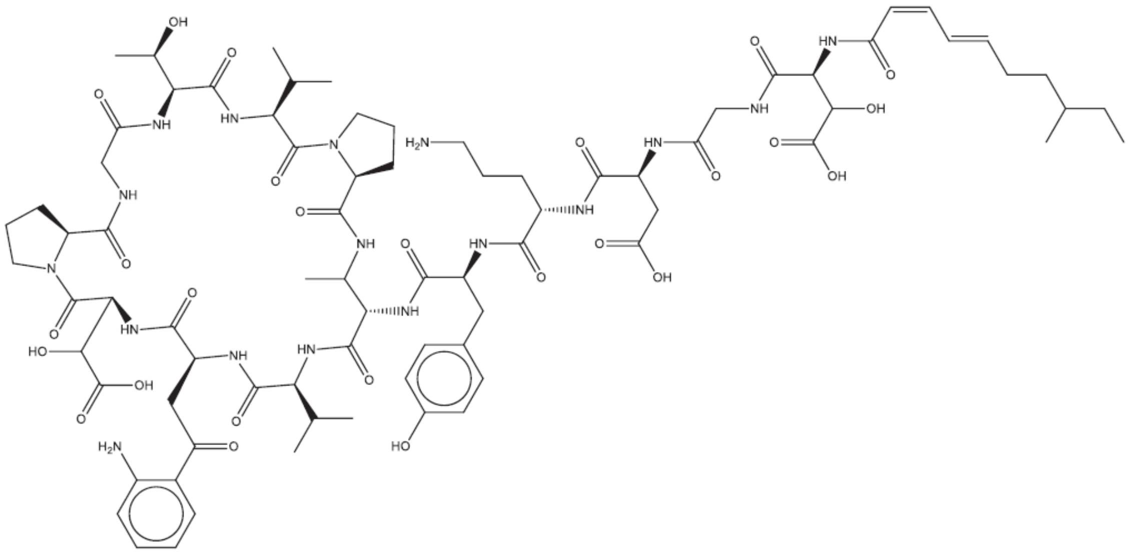

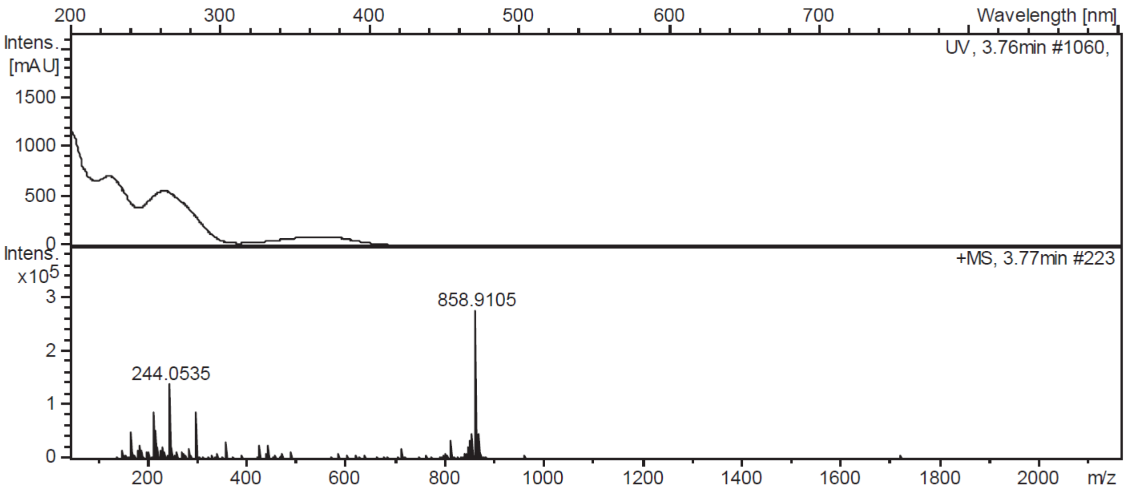

Section 3.3. Effect of VOCs in multiple co-culture

CS065a:

Alteramide A, $C_{29}H_{38}N_2O_6$:

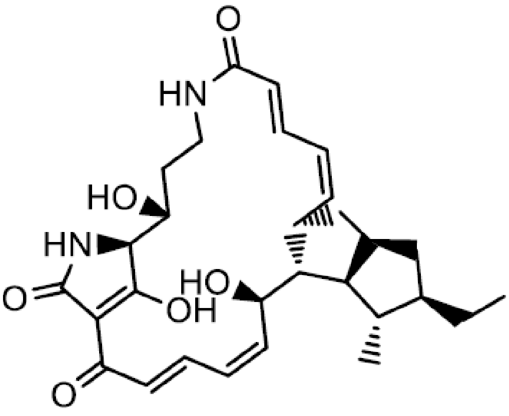

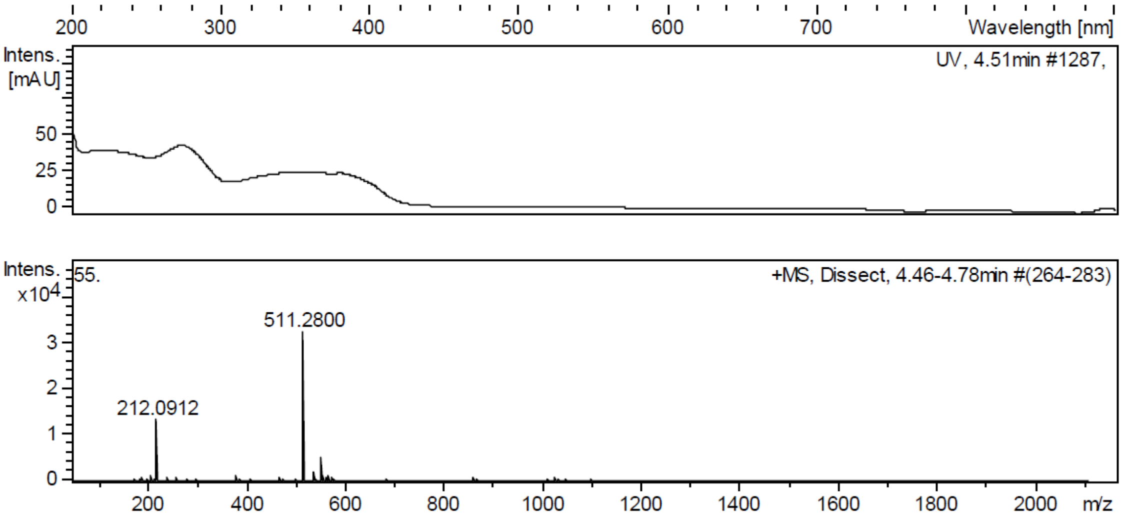

Chromomycin A3, $C_{57}H_{82}O_{26}$:

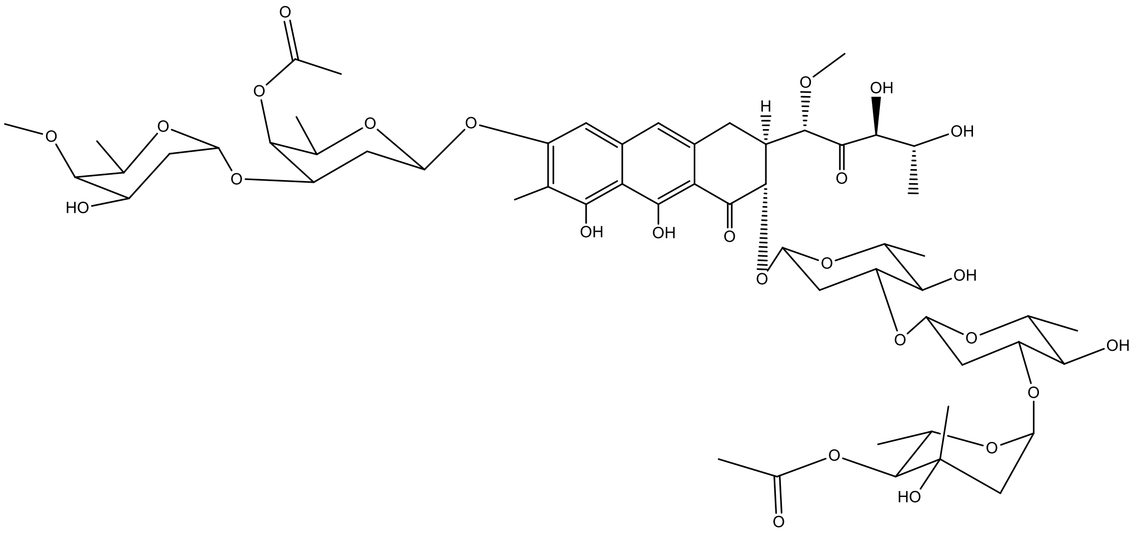

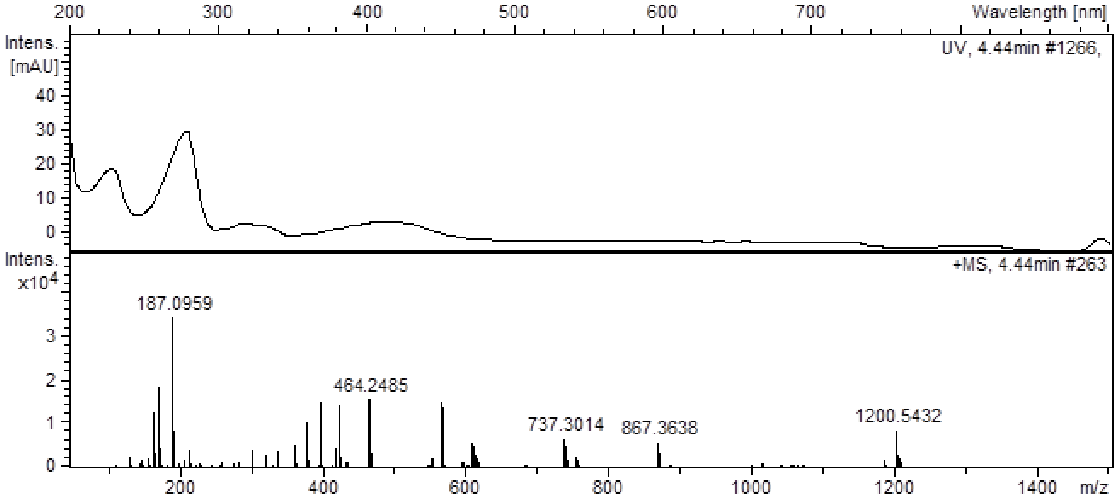

Overview of the dereplication analysis.

Compound	Molecular formula	Measured mass	Calculated mass	Most prominent ions	UV max (nm)
Collismycin A-B	$C_{13}H_{13}N_3O_2S$	276.0821	275.33	276.0821<br>605.0723	249,5<br>332,7<br>469,8
Collismycin C	$C_{13}H_{14}N_2O_2S$	263.0850	262.33	263.0850	225,0<br>249,5<br>332,7<br>469,8

Collismycin D	$C_{13}H_{11}N_3OS$	258.0696	257.32	258.0696<br>574.1917	225,0<br>332,7<br>469,8
Granaticin A	$C_{22}H_{20}O_{10}$	445.1136	444.39	276.0801<br>445.1136<br>906.2452	215,3<br>280,9<br>494,2
Granaticin C	$C_{28}H_{30}O_{12}$	576.2074	558.54	276.0802<br>445.1135<br>576.2074<br>855.2933	215,3<br>280,9<br>494,2
Cosmomycin D	$C_{60}H_{88}N_2O_{22}$	1189.5913	1189.36	595.2984<br>1189.5913	235,4<br>255,0<br>295,0<br>493,5
Cycloheximide	$C_{15}H_{23}NO_4$	282.1737	281.35	282.1737<br>585.3142	200,0
Actiphenol	$C_{15}H_{17}NO_4$	276.1234	275.30	165.0912<br>276.1234	216,5<br>262, 4<br>345,2
Maltophilin	$C_{29}H_{38}N_2O_6$	511.2804	510.63	511.2804	222,6<br>322,8
Alteramide A	$C_{29}H_{38}N_2O_6$	511.2800	510.63	212.0912<br>511.2800	273,5<br>360,1
Piperazinedione XR 334	$C_{19}H_{16}N_2O_3$	321.1232	320.35	321.1232<br>641.2390	241,0<br>334,6
Piperazinedione XR 330	$C_{20}H_{18}N_2O_3$	335.1391	334.38	335.1391<br>669.2708	241,0<br>334,6
Rumycin 1	$C_{78}H_{111}N_{17}O_{26}$	851.9025	1702.84	151.1116<br>282.1333<br>851.9025	231,2<br>264,4<br>365,1
Rumycin 2	$C_{79}H_{113}N_{17}O_{26}$	858.9105	1716.87	244.0535<br>858.9105	226,9<br>261.8<br>365,7
Chromomycin A3	$C_{57}H_{82}O_{26}$	1200.5432	1183.26	187.0959<br>464.2485<br>737.3014<br>867.3638<br>1200.5432	229,3<br>277,8<br>317,3<br>403.7
