# Supplementary material for: Volatile Compounds in Actinomycete Communities: A New Tool for Biosynthetic Gene Cluster Activation, Cooperative Growth Promotion, and Drug Discovery
Source: Cells. 2022 Nov 5;11(21):3510. doi: 10.3390/cells11213510 (PMC9655753; doi:10.3390/cells11213510)
Supplement: Supplementary file 1 [file cells-11-03510-s001.zip › Table S19. rmc gene cluster.pdf]

Table S19. Predicted function of *rmc* genes.

Gene	Function	Closest homologue	
rmc1	diaminobutyric acid synthase C	*Streptomyces viridochromogenes*	AEG64688.1
rmc2	argininosuccinate lyase	*Streptomyces globisporus*	MCC8479050.1
rmc3	pyridoxal-phosphate dependent enzyme (cysteine synthetase)	*Streptomyces globisporus*	MCC8479049.1
rmc4	NRPS	*Streptomyces* sp. CAI-121	NUV71303.1
rmc5	Kinase	*Streptomyces badius*	WP_199887694.1
rmc6	TauD/TfdA family dioxygenase	Multispecies - *Streptomyces*	WP_069738900.1
rmc7	tryptophan 2,3-dioxygenase family protein	Multispecies - *Streptomyces*	WP_079149654.1
rmc8	fatty acyl-AMP ligase	*Streptomyces globisporus*	MCC8479045.1
rmc9	acyl-CoA dehydrogenase family protein	*Streptomyces globisporus*	MCC8479044.1
rmc10	acyl-CoA dehydrogenase	*Streptomyces globisporus*	MCC8479043.1
rmc11	acyl carrier protein	*Streptomyces globisporus*	MCC8479042.1
rmc12	NRPS	*Streptomyces badius*	WP_199887692.1
rmc13	NRPS	*Streptomyces globisporus*	MCC8482945.1
rmc14	NRPS	*Streptomyces globisporus*	MCC8482950.1
rmc15	NRPS	*Streptomyces globisporus*	MCC8482893.1
rmc16	NRPS	*Streptomyces globisporus*	MCC8482894.1
rmc17	NRPS	*Streptomyces globisporus*	MCC8482950.1
rmc18	NRPS	*Streptomyces globisporus*	MCC8482386.1
rmc19	NRPS	*Streptomyces globisporus*	MCC8482387.1
rmc20	MbtH family protein	*Streptomyces badius*	WP_199889602.1
rmc21	DedA family protein	*Streptomyces parvus*	WP_148904607.1
rmc22	ABC transporter ATP-binding protein	*Streptomyces parvus*	WP_148904605.1
rmc23	ABC transporter permease subunit	*Streptomyces* sp. CAI 127	WP_175515771.1
rmc24	response regulator transcription factor (LuxR)	Multispecies - *Streptomyces*	WP_176735228.1
rmc25	TauD/TfdA family dioxygenase	Multispecies - *Streptomyces*	WP_069737015.1
rmc26	Signal transduction histidine kinase	*Streptomyces* sp. CAI 127	NUW01750.1
rmc27	alpha/beta hydrolase	*Streptomyces* sp. CAI 127	WP_175515773.1
rmc28	ABC transporter permease	*Streptomyces* sp. CAI 127	WP_175515774.1
rmc29	ATP-binding cassette domain-containing protein	*Streptomyces* sp. CAI 127	WP_175515775.1
rmc30	SCO5389 family protein	*Streptomyces* sp. CB02366	WP_073763754.1
rmc31	LLM class flavin-dependent oxidoreductase	*Streptomyces* sp. CAI 127	WP_175515776.1
rmc32	ATP/GTP-binding protein	*Streptomyces globisporus*	WP_118906321.1
rmc33	ABC transporter permease subunit	*Streptomyces globisporus*	WP_118906320.1
rmc34	ATP-binding cassette domain-containing protein	*Streptomyces* sp. SID4931	MYV62918.1
rmc35	ABC transporter permease subunit	*Streptomyces* sp. SID14515	WP_164612903.1
rmc36	ABC transporter ATP-binding protein	*Streptomyces filamentosus* NRRL 15998	EFE77641.1
