# Supplementary material for: Volatile Compounds in Actinomycete Communities: A New Tool for Biosynthetic Gene Cluster Activation, Cooperative Growth Promotion, and Drug Discovery
Source: Cells. 2022 Nov 5;11(21):3510. doi: 10.3390/cells11213510 (PMC9655753; doi:10.3390/cells11213510)
Supplement: Supplementary file 1 [file cells-11-03510-s001.zip › Tables S1-S13. Dual co-cultures.pdf]

Table S1. Overview of the dual co-culture against *Streptomyces* sp. CS014.

Strain	Growth medium	Outcomes in *Streptomyces* sp. CS014
CS057	SFM	No effects observed
	YMA	Increased bioactivity against *M. luteus* (collismycin and granaticin)[1]
	R5A	No effects observed
CS065a	SFM	No effects observed
	YMA	No effects observed
	R5A	No effects observed
CS081a	SFM	No effects observed
	YMA	Increased bioactivity against *M. luteus* (collismycin and granaticin)[1]
	R5A	No effects observed
CS090a	SFM	No effects observed
	YMA	Increased bioactivity against *M. luteus* (collismycin and granaticin)[1]
	R5A	No effects observed
CS113	SFM	No effects observed
	YMA	No effects observed
	R5A	No effects observed
CS131	SFM	No effects observed
	YMA	Increased bioactivity against *M. luteus* (collismycin and granaticin)[1]
	R5A	No effects observed
CS147	SFM	No effects observed
	YMA	No effects observed
	R5A	No effects observed
CS149	SFM	No effects observed
	YMA	Increased bioactivity against *M. luteus* (collismycin and granaticin)[1]
	R5A	No effects observed
CS159	SFM	No effects observed
	YMA	No effects observed
	R5A	No effects observed
CS194	SFM	No effects observed
	YMA	No effects observed
	R5A	No effects observed
CS207	SFM	No effects observed
	YMA	No effects observed
	R5A	No effects observed
CS227	SFM	No effects observed
	YMA	No effects observed
	R5A	No effects observed

[1] Collismycin was only produced in coculture. Higher granaticin production in coculture compared to monoculture.

Table S2. Overview of the dual co-culture against *Streptomyces* sp. CS057.

Strain	Growth medium	Outcomes in *Streptomyces* sp. CS057
CS014	SFM	No effects observed
	YMA	No effects observed
	R5A	No effects observed
CS065a	SFM	No effects observed
	YMA	No effects observed
	R5A	No effects observed
CS081a	SFM	No effects observed
	YMA	Increased bioactivity against *M. luteus* and *E. weberi* (cycloheximide and actiphenol)[1]
	R5A	No effects observed
CS090a	SFM	No effects observed
	YMA	No effects observed
	R5A	No effects observed
CS113	SFM	No effects observed
	YMA	No effects observed
	R5A	No effects observed
CS131	SFM	No effects observed
	YMA	No effects observed
	R5A	No effects observed
CS147	SFM	No effects observed
	YMA	No effects observed
	R5A	No effects observed
CS149	SFM	No effects observed
	YMA	No effects observed
	R5A	No effects observed
CS159	SFM	No effects observed
	YMA	Slight increased bioactivity against *M. luteus* and *E. weberi* (cycloheximide and actiphenol)[1]
	R5A	No effects observed
CS194	SFM	No effects observed
	YMA	No effects observed
	R5A	No effects observed
CS207	SFM	No effects observed
	YMA	No effects observed
	R5A	No effects observed
CS227	SFM	No effects observed
	YMA	No effects observed
	R5A	No effects observed

[1] Only traces of cycloheximde and actiphenol were detected in monoculture.

Table S3. Overview of the dual co-culture against *Streptomyces* sp. CS065a.

Strain	Growth medium	Outcomes in *Streptomyces* sp. CS065a
CS014	SFM	No effects observed
	YMA	No effects observed
	R5A	No effects observed
CS057	SFM	No effects observed
	YMA	No effects observed
	R5A	No effects observed
CS081a	SFM	No effects observed
	YMA	No effects observed
	R5A	No effects observed
CS090a	SFM	No effects observed
	YMA	No effects observed
	R5A	No effects observed
CS113	SFM	No effects observed
	YMA	No effects observed
	R5A	No effects observed
CS131	SFM	No effects observed
	YMA	No effects observed
	R5A	No effects observed
CS147	SFM	No effects observed
	YMA	No effects observed
	R5A	No effects observed
CS149	SFM	No effects observed
	YMA	No effects observed
	R5A	No effects observed
CS159	SFM	No effects observed
	YMA	No effects observed
	R5A	No effects observed
CS194	SFM	No effects observed
	YMA	No effects observed
	R5A	No effects observed
CS207	SFM	No effects observed
	YMA	No effects observed
	R5A	No effects observed
CS227	SFM	No effects observed
	YMA	No effects observed
	R5A	No effects observed

Table S4. Overview of the dual co-culture against *Streptomyces* sp. CS081a.

Strain	Growth medium	Outcomes in *Streptomyces* sp. CS081a
CS014	SFM	No effects observed
	YMA	Increased bioactivity against *M. luteus* (cosmomycins)[1]
	R5A	No effects observed
CS057	SFM	No effects observed
	YMA	No effects observed
	R5A	No effects observed
CS065a	SFM	No effects observed
	YMA	No effects observed
	R5A	No effects observed
CS090a	SFM	No effects observed
	YMA	No effects observed
	R5A	No effects observed
CS113	SFM	No effects observed
	YMA	No effects observed
	R5A	No effects observed
CS131	SFM	No effects observed
	YMA	No effects observed
	R5A	No effects observed
CS147	SFM	No effects observed
	YMA	No effects observed
	R5A	No effects observed
CS149	SFM	No effects observed
	YMA	No effects observed
	R5A	No effects observed
CS159	SFM	No effects observed
	YMA	Slight increased bioactivity against *M. luteus* (cosmomycins)[1]
	R5A	No effects observed
CS194	SFM	No effects observed
	YMA	No effects observed
	R5A	No effects observed
CS207	SFM	No effects observed
	YMA	No effects observed
	R5A	No effects observed
CS227	SFM	No effects observed
	YMA	No effects observed
	R5A	No effects observed

[1] Cosmomycins were only produced in coculture.

Table S5. Overview of the dual co-culture against *Streptomyces* sp. CS090a.

Strain	Growth medium	Outcomes in *Streptomyces* sp. CS090a
CS014	SFM	No effects observed
	YMA	No effects observed
	R5A	No effects observed
CS057	SFM	No effects observed
	YMA	Bioactivity against *E. weberi* (maltophilins and alteramides)[1]
	R5A	No effects observed
CS065a	SFM	No effects observed
	YMA	No effects observed
	R5A	No effects observed
CS081a	SFM	No effects observed
	YMA	Bioactivity against *E. weberi* (maltophilins and alteramides)[1]
	R5A	No effects observed
CS113	SFM	No effects observed
	YMA	No effects observed
	R5A	No effects observed
CS131	SFM	No effects observed
	YMA	No effects observed
	R5A	No effects observed
CS147	SFM	No effects observed
	YMA	No effects observed
	R5A	No effects observed
CS149	SFM	No effects observed
	YMA	No effects observed
	R5A	No effects observed
CS159	SFM	No effects observed
	YMA	No effects observed
	R5A	No effects observed
CS194	SFM	No effects observed
	YMA	No effects observed
	R5A	No effects observed
CS207	SFM	No effects observed
	YMA	No effects observed
	R5A	No effects observed
CS227	SFM	No effects observed
	YMA	No effects observed
	R5A	No effects observed

[1] Maltophilins and alteramides were only produced in coculture.

Table S6. Overview of the dual co-culture against *Streptomyces* sp. CS113.

Strain	Growth medium	Outcomes in *Streptomyces* sp. CS113
CS014	SFM	No effects observed
	YMA	No effects observed
	R5A	No effects observed
CS057	SFM	No effects observed
	YMA	No effects observed
	R5A	No effects observed
CS065a	SFM	No effects observed
	YMA	No effects observed
	R5A	No effects observed
CS081a	SFM	No effects observed
	YMA	No effects observed
	R5A	No effects observed
CS090a	SFM	No effects observed
	YMA	No effects observed
	R5A	No effects observed
CS131	SFM	No effects observed
	YMA	No effects observed
	R5A	No effects observed
CS147	SFM	No effects observed
	YMA	No effects observed
	R5A	No effects observed
CS149	SFM	No effects observed
	YMA	No effects observed
	R5A	No effects observed
CS159	SFM	No effects observed
	YMA	No effects observed
	R5A	No effects observed
CS194	SFM	No effects observed
	YMA	No effects observed
	R5A	No effects observed
CS207	SFM	No effects observed
	YMA	No effects observed
	R5A	No effects observed
CS227	SFM	No effects observed
	YMA	No effects observed
	R5A	No effects observed

Table S7. Overview of the dual co-culture against *Streptomyces* sp. CS131.

Strain	Growth medium	Outcomes in *Streptomyces* sp. CS131
CS014	SFM	No effects observed
	YMA	No effects observed
	R5A	No effects observed
CS057	SFM	No effects observed
	YMA	No effects observed
	R5A	No effects observed
CS065a	SFM	No effects observed
	YMA	No effects observed
	R5A	No effects observed
CS081a	SFM	No effects observed
	YMA	No effects observed
	R5A	No effects observed
CS090a	SFM	No effects observed
	YMA	No effects observed
	R5A	No effects observed
CS113	SFM	No effects observed
	YMA	No effects observed
	R5A	No effects observed
CS147	SFM	No effects observed
	YMA	No effects observed
	R5A	No effects observed
CS149	SFM	No effects observed
	YMA	No effects observed
	R5A	No effects observed
CS159	SFM	No effects observed
	YMA	No effects observed
	R5A	No effects observed
CS194	SFM	No effects observed
	YMA	No effects observed
	R5A	No effects observed
CS207	SFM	No effects observed
	YMA	No effects observed
	R5A	No effects observed
CS227	SFM	No effects observed
	YMA	No effects observed
	R5A	No effects observed

Table S8. Overview of the dual co-culture against *Streptomyces* sp. CS147.

Strain	Growth medium	Outcomes in *Streptomyces* sp. CS147
CS014	SFM	No effects observed
	YMA	No effects observed
	R5A	No effects observed
CS057	SFM	No effects observed
	YMA	No effects observed
	R5A	No effects observed
CS065a	SFM	No effects observed
	YMA	No effects observed
	R5A	No effects observed
CS081a	SFM	No effects observed
	YMA	No effects observed
	R5A	No effects observed
CS090a	SFM	No effects observed
	YMA	No effects observed
	R5A	No effects observed
CS113	SFM	No effects observed
	YMA	No effects observed
	R5A	No effects observed
CS131	SFM	No effects observed
	YMA	No effects observed
	R5A	No effects observed
CS149	SFM	No effects observed
	YMA	No effects observed
	R5A	No effects observed
CS159	SFM	No effects observed
	YMA	No effects observed
	R5A	No effects observed
CS194	SFM	No effects observed
	YMA	No effects observed
	R5A	No effects observed
CS207	SFM	No effects observed
	YMA	No effects observed
	R5A	No effects observed
CS227	SFM	No effects observed
	YMA	No effects observed
	R5A	No effects observed

Table S9. Overview of the dual co-culture against *Streptomyces* sp. CS149.

Strain	Growth medium	Outcomes in *Streptomyces* sp. CS149
CS014	SFM	No effects observed
	YMA	No effects observed
	R5A	No effects observed
CS057	SFM	No effects observed
	YMA	No effects observed
	R5A	No effects observed
CS065a	SFM	No effects observed
	YMA	No effects observed
	R5A	No effects observed
CS081a	SFM	Bioactivity against *M. luteus* (rumycins)[1]
	YMA	No effects observed
	R5A	No effects observed
CS090a	SFM	No effects observed
	YMA	No effects observed
	R5A	No effects observed
CS113	SFM	No effects observed
	YMA	No effects observed
	R5A	No effects observed
CS131	SFM	Bioactivity against *M. luteus* (rumycins)[1]
	YMA	No effects observed
	R5A	No effects observed
CS147	SFM	No effects observed
	YMA	No effects observed
	R5A	No effects observed
CS159	SFM	No effects observed
	YMA	No effects observed
	R5A	No effects observed
CS194	SFM	No effects observed
	YMA	No effects observed
	R5A	No effects observed
CS207	SFM	No effects observed
	YMA	No effects observed
	R5A	No effects observed
CS227	SFM	No effects observed
	YMA	No effects observed
	R5A	No effects observed

[1] Rumycins were only produced in coculture.

Table S10. Overview of the dual co-culture against *Streptomyces* sp. CS159.

Strain	Growth medium	Outcomes in *Streptomyces* sp. CS159
CS014	SFM	No effects observed
	YMA	No effects observed
	R5A	No effects observed
CS057	SFM	No effects observed
	YMA	No effects observed
	R5A	No effects observed
CS065a	SFM	No effects observed
	YMA	No effects observed
	R5A	No effects observed
CS081a	SFM	No effects observed
	YMA	No effects observed
	R5A	No effects observed
CS090a	SFM	No effects observed
	YMA	No effects observed
	R5A	No effects observed
CS113	SFM	No effects observed
	YMA	No effects observed
	R5A	No effects observed
CS131	SFM	No effects observed
	YMA	No effects observed
	R5A	No effects observed
CS147	SFM	No effects observed
	YMA	No effects observed
	R5A	No effects observed
CS149	SFM	No effects observed
	YMA	No effects observed
	R5A	No effects observed
CS194	SFM	No effects observed
	YMA	No effects observed
	R5A	No effects observed
CS207	SFM	No effects observed
	YMA	No effects observed
	R5A	No effects observed
CS227	SFM	No effects observed
	YMA	No effects observed
	R5A	No effects observed

Table S11. Overview of the dual co-culture against *Streptomyces* sp. CS194.

Strain	Growth medium	Outcomes in *Streptomyces* sp. CS194
CS014	SFM	Bioactivity against *M. luteus* (piperazinediones?)[1]
	YMA	No effects observed
	R5A	No effects observed
CS057	SFM	Bioactivity against *M. luteus* (piperazinediones?)[1]
	YMA	No effects observed
	R5A	No effects observed
CS065a	SFM	No effects observed
	YMA	No effects observed
	R5A	No effects observed
CS081a	SFM	Bioactivity against *M. luteus* (piperazinediones?)[1]
	YMA	No effects observed
	R5A	No effects observed
CS090a	SFM	No effects observed
	YMA	No effects observed
	R5A	No effects observed
CS113	SFM	Difference in the timing of the sporulation
	YMA	No effects observed
	R5A	No effects observed
CS131	SFM	Difference in the timing of the sporulation
	YMA	No effects observed
	R5A	No effects observed
CS147	SFM	Difference in the timing of the sporulation
	YMA	No effects observed
	R5A	No effects observed
CS149	SFM	No effects observed
	YMA	No effects observed
	R5A	No effects observed
CS159	SFM	No effects observed
	YMA	No effects observed
	R5A	No effects observed
CS207	SFM	No effects observed
	YMA	No effects observed
	R5A	No effects observed
CS227	SFM	Difference in the timing of the sporulation and bioactivity against *M. luteus* (piperazinediones?)[1]
	YMA	No effects observed
	R5A	No effects observed

[1] It was not possible to make a comparative study of the metabolites produced by CS194 grown in monoculture vs. coculture because the strain did not grow in monoculture. Bioactive piperazinediones were detected in coculture samples and their involvement in the observed bioactivity should be taken as a hypothesis.

Table S12. Overview of the dual co-culture against *Streptomyces* sp. CS207.

Strain	Growth medium	Outcomes in *Streptomyces* sp. CS207
CS014	SFM	No effects observed
	YMA	Increased bioactivity against *E. coli* and *M. luteus*
	R5A	No effects observed
CS057	SFM	No effects observed
	YMA	Increased bioactivity against *E. coli* and *M. luteus*
	R5A	No effects observed
CS065a	SFM	No effects observed
	YMA	No effects observed
	R5A	No effects observed
CS081a	SFM	No effects observed
	YMA	Increased bioactivity against *E. coli* and *M. luteus*
	R5A	No effects observed
CS090a	SFM	No effects observed
	YMA	No effects observed
	R5A	No effects observed
CS113	SFM	No effects observed
	YMA	No effects observed
	R5A	No effects observed
CS131	SFM	No effects observed
	YMA	No effects observed
	R5A	No effects observed
CS147	SFM	No effects observed
	YMA	No effects observed
	R5A	No effects observed
CS149	SFM	No effects observed
	YMA	No effects observed
	R5A	No effects observed
CS159	SFM	No effects observed
	YMA	No effects observed
	R5A	No effects observed
CS194	SFM	No effects observed
	YMA	No effects observed
	R5A	No effects observed
CS227	SFM	No effects observed
	YMA	Increased bioactivity against *E. coli* and *M. luteus*
	R5A	No effects observed

Table S13. Overview of the dual co-culture against *Streptomyces* sp. CS227.

Strain	Growth medium	Outcomes in *Streptomyces* sp. CS227
CS014	SFM	No effects observed
	YMA	No effects observed
	R5A	No effects observed
CS057	SFM	No effects observed
	YMA	No effects observed
	R5A	No effects observed
CS065a	SFM	No effects observed
	YMA	No effects observed
	R5A	No effects observed
CS081a	SFM	No effects observed
	YMA	No effects observed
	R5A	No effects observed
CS090a	SFM	No effects observed
	YMA	No effects observed
	R5A	No effects observed
CS113	SFM	No effects observed
	YMA	No effects observed
	R5A	No effects observed
CS131	SFM	No effects observed
	YMA	No effects observed
	R5A	No effects observed
CS147	SFM	No effects observed
	YMA	No effects observed
	R5A	No effects observed
CS149	SFM	No effects observed
	YMA	No effects observed
	R5A	No effects observed
CS159	SFM	No effects observed
	YMA	No effects observed
	R5A	No effects observed
CS194	SFM	No effects observed
	YMA	No effects observed
	R5A	No effects observed
CS207	SFM	No effects observed
	YMA	No effects observed
	R5A	No effects observed
