# Supplementary material for: Volatile Compounds in Actinomycete Communities: A New Tool for Biosynthetic Gene Cluster Activation, Cooperative Growth Promotion, and Drug Discovery
Source: Cells. 2022 Nov 5;11(21):3510. doi: 10.3390/cells11213510 (PMC9655753; doi:10.3390/cells11213510)
Supplement: Supplementary file 1 [file cells-11-03510-s001.zip › Tables S14-S18. Multiple co-cultures.pdf]

Table S14. Overview of the multiple co-culture against *Streptomyces* sp. CS065a.

Strains	Growth medium	Outcomes in *Streptomyces* sp. CS065a
CS147, CS207, CS113	SFM	Increased bioactivity against *M. luteus* (alteramide, chromomycin) and bioactivity against *E. coli*
	YMA	Increased bioactivity against *M. luteus* (alteramide, chromomycin) and *E. coli*
CS147, CS207, CS090a	SFM	Increased bioactivity against *M. luteus* (alteramide, chromomycin) and bioactivity against *E. coli*
	YMA	Increased bioactivity against *M. luteus* (alteramide, chromomycin) and *E. coli*
CS147, CS090a, CS113	SFM	No effects observed
	YMA	Increased bioactivity against *M. luteus* (alteramide, chromomycin) and showed bioactivity against *E. coli*
CS090a, CS207, CS113	SFM	Increased bioactivity against *M. luteus* (alteramide, chromomycin) and showed bioactivity against *E. coli*
	YMA	Increased bioactivity against *M. luteus* (alteramide, chromomycin) and *E. coli*

Table S15. Overview of the multiple co-culture against *Streptomyces* sp. CS090a.

Strains	Growth medium	Outcomes in *Streptomyces* sp. CS090a
CS147, CS207, CS113	SFM	No effects observed
	YMA	No effects observed
CS147, CS207, CS065a	SFM	No effects observed
	YMA	No effects observed
CS147, CS065a, CS113	SFM	No effects observed
	YMA	No effects observed
CS065a,CS207, CS113	SFM	No effects observed
	YMA	No effects observed

Table S16. Overview of the multiple co-culture against *Streptomyces* sp. CS113.

Strains	Growth medium	Outcomes in *Streptomyces* sp. CS113
CS147, CS207, CS090a	SFM	No effects observed
	YMA	No effects observed
CS147, CS207, CS065a	SFM	No effects observed
	YMA	No effects observed
CS147, CS065a, CS090a	SFM	No effects observed
	YMA	No effects observed
CS065a,CS207, CS090a	SFM	No effects observed
	YMA	No effects observed

Table S17. Overview of the multiple co-culture against *Streptomyces* sp. CS147.

Strains	Growth medium	Outcomes in *Streptomyces* sp. CS147
CS113, CS207, CS090a	SFM	No effects observed
	YMA	No effects observed
CS113, CS207, CS065a	SFM	No effects observed
	YMA	No effects observed
CS113, CS065a, CS090a	SFM	No effects observed
	YMA	No effects observed
CS065a,CS207, CS090a	SFM	No effects observed
	YMA	No effects observed

Table S18. Overview of the multiple co-culture against *Streptomyces* sp. CS207.

Strains	Growth medium	Outcomes in *Streptomyces* sp. CS207
CS113, CS147, CS090a	SFM	No effects observed
	YMA	No effects observed
CS113, CS147, CS065a	SFM	No effects observed
	YMA	No effects observed
CS113, CS065a, CS090a	SFM	No effects observed
	YMA	No effects observed
CS065a,CS147, CS090a	SFM	No effects observed
	YMA	No effects observed
