# Supplementary figures and images for: Volatile Compounds in Actinomycete Communities: A New Tool for Biosynthetic Gene Cluster Activation, Cooperative Growth Promotion, and Drug Discovery
Source: Cells. 2022 Nov 5;11(21):3510. doi: 10.3390/cells11213510 (PMC9655753; doi:10.3390/cells11213510)

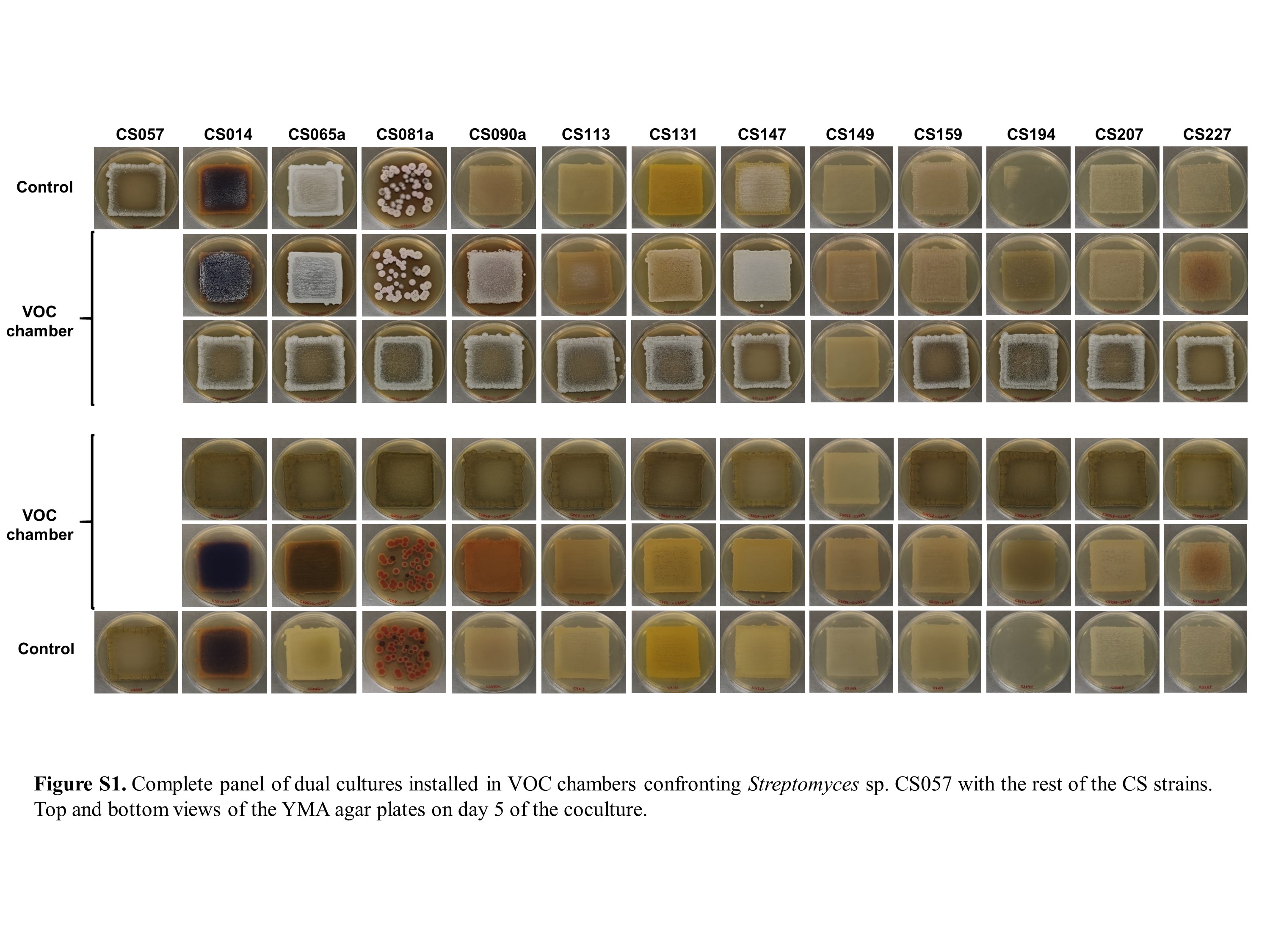

Supplement: Supplementary file 1 [file cells-11-03510-s001.zip › Figure S1. Complete panel CS057.jpg]

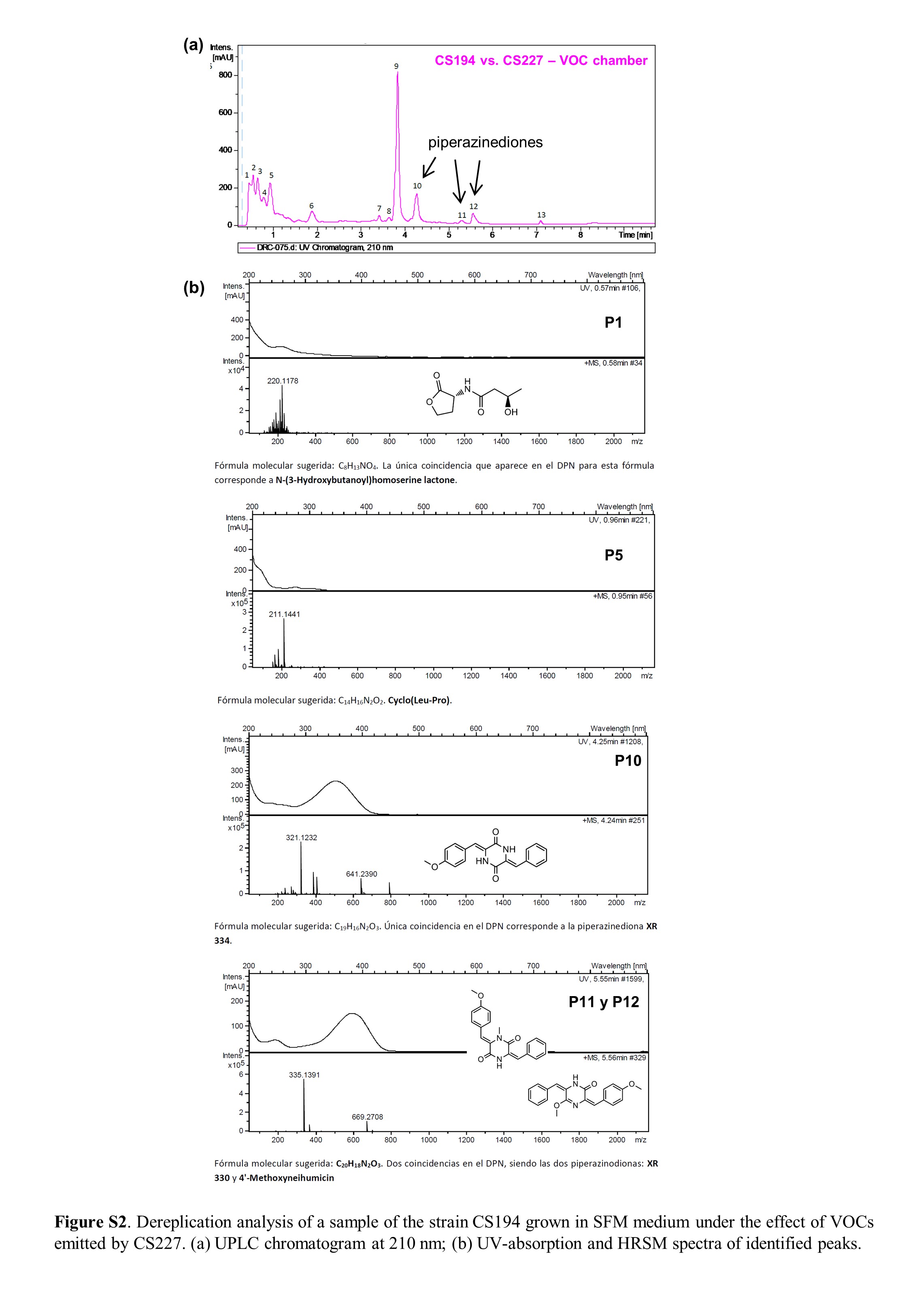

Supplement: Supplementary file 1 [file cells-11-03510-s001.zip › Figure S2. Dereplication CS194.jpg]
